# Hydrodynamic evaluation of a spider-inspired underwater robot using distributed flapping fin propulsion

**DOI:** 10.1038/s41598-026-57795-6

**Published:** 2026-06-13

**Authors:** Vishnu G. Nair, Rithvik Marneni, B. Gowrava Shenoy, Lung-Jieh Yang, Chandrashekhar Tasupalli, Mohammad Zuber, Spoorthi Singh

**Affiliations:** 1 Manipal Institute of Technology, Manipal Academy of Higher Education, Manipal, India; 2 Department of Mechanical and Electromechanical Engineering, Tamkang University, New Taipei City, 251 Taiwan

**Keywords:** Bio-inspired flapping-fin propulsion, Underwater hexapod robot, Unsteady hydrodynamics, Computational fluid dynamics (CFD), Strouhal and Reynolds number scaling, Engineering, Mathematics and computing, Physics

## Abstract

This paper presents the design and hydrodynamic evaluation of a spider-inspired underwater hexapod robot employing distributed flapping-fin propulsion. Unlike conventional bio-inspired underwater robots with centralized fin actuation, the proposed system integrates flexible bilateral side fins between leg triads, enabling decentralized lift generation and inherent body stabilization. A three-tier validation framework is adopted, comprising (i) quasi-steady analytical estimation of lift, drag, Reynolds number, and lift-to-power scaling, (ii) transient Computational Fluid Dynamics (CFD) simulations in ANSYS Fluent resolving pressure distribution, vortex shedding, and wake evolution, and (iii) preliminary experimental validation using a laboratory-scale prototype in controlled water-tank conditions. Theoretical, numerical, and experimental results show close agreement, with lift predictions within ± 10–15% across operating regimes. CFD results indicate stable hydrodynamic performance over fin-tip velocities U = 0.6–0.8 m/s, yielding a nearly constant lift-to-drag ratio of ≈ 1.3. Non-dimensional analysis confirms operation within an efficient Strouhal number range. The study establishes the hydrodynamic feasibility and energetic advantages of distributed flapping-fin propulsion integrated with a multi-legged body architecture. The results provide validated design insights for the development of manoeuvrable and energy-efficient underwater robotic platforms for inspection, monitoring, and exploration tasks.

## Introduction

Propulsive systems based on flapping fins have attracted increasing attention in underwater robotics due to their ability to generate thrust and manoeuvring forces through unsteady hydrodynamic mechanisms. Unlike conventional propellers, flapping fins exploit time-varying lift, vortex shedding, and fluid–structure interaction (FSI) to produce propulsion, similar to natural swimming observed in rays and fish^1,2^. Such systems typically operate in transitional-to-turbulent flow regimes, where performance is governed by Reynolds and Strouhal number scaling^3–5^. Prior studies on bio-inspired underwater robots, including pectoral-fin-driven platforms modelled after bluegill sunfish, have demonstrated that flexible fin structures enable effective thrust and lateral manoeuvring through controlled unsteady flow interactions^6^. Similarly, experimental and numerical investigations on manta-bots and caudal-fin swimmers have shown that vortex dynamics and fin compliance play a key role in lift enhancement and propulsive efficiency^2,7^. Advances in computational modelling have enabled fully coupled FSI simulations using partitioned solvers, allowing accurate resolution of fin deformation and vortex-induced force generation^8,9^. In parallel, analytical approaches based on quasi-steady force models and Strouhal number scaling (typically St ≈ 0.2–0.4) have provided useful benchmarks for evaluating thrust and efficiency across flapping regimes^3,10,11^. While numerous engineered platforms including robotic fish^12^, remora-inspired adhesion systems^13^, and recent bio-hybrid swimmers^14^, demonstrate the effectiveness of fin-based propulsion, most rely on centralized actuation or limited fin configurations. As a result, distributed propulsion and inherent body stabilization remain underexplored, particularly in multi-bodied robotic architectures. Addressing this gap, the present work introduces a spider-inspired underwater hexapod integrating bilateral flapping fins between leg triads to achieve distributed thrust generation and hydrodynamic stabilization.

Recent developments in biomimetic underwater propulsion have highlighted the roles of structural compliance, coordinated control, and distributed actuation in improving thrust generation and manoeuvrability. Zhong et al. demonstrated that a wire-driven robotic fish with a compliant body and tail achieves enhanced propulsive efficiency under unsteady flow conditions^15^. Barbera et al. showed that coordinated pectoral-fin actuation can effectively stabilize yaw and pitch through active attitude control strategies^16^. The influence of wake dynamics has also been investigated by Wang and Zhang, who applied fuzzy vorticity control to a lunate flapping tail, demonstrating that controlled exploitation of vortex shedding can increase thrust output^17^. Distributed actuation has further been explored using alternative actuation technologies; for example, Yang et al. employed IPMC artificial muscles to drive both pectoral and caudal fins, achieving improved manoeuvrability through decentralized control^18^. High-agility fin-based propulsion has been demonstrated by Ng et al. with the SNAPP robotic fish, capable of rapid three-dimensional manoeuvres using coordinated flapping fins^19^. Complementary experimental studies by Shao et al. confirmed that deformable caudal fins significantly enhance thrust performance, reinforcing the benefits of fin compliance for efficient aquatic locomotion^20^. Beyond fish-like swimmers, recent investigations into undulating fin propulsion have shown that parameters such as fin aspect ratio, oscillation frequency, and wave kinematics strongly influence thrust and efficiency, as validated through combined CFD and experimental studies^21^. Integrated bio-inspired platforms, including the bionic mantis shrimp robot, further illustrate the importance of compact actuation and control architectures for effective underwater manoeuvring and depth regulation^22^. Collectively, these studies emphasize the emerging convergence of hydrodynamics, control, and distributed actuation in bio-inspired underwater robotics, directly motivating the distributed flapping-fin approach adopted in the present spider-inspired design.

Despite advances in bio-inspired underwater robotics, most existing designs rely on monolithic fins or centralized actuation, limiting distributed thrust generation and inherent body stabilization. Robotic fish inspired by sunfish, rays, and manta-bots typically concentrate propulsion in a single pectoral or caudal fin system, providing manoeuvrability but reduced redundancy under unsteady flow conditions^2,4,6,7,12^. Even recent bio-hybrid platforms employing flexible fins remain constrained by centralized architectures^14^. This highlights the need for underwater robots that decentralize propulsion across multiple body segments. Addressing this gap, the present work introduces a spider-inspired underwater hexapod integrating bilateral flexible side fins to achieve distributed propulsion and hydrodynamic stabilization through coordinated multi-fin actuation. Most bio-inspired underwater robots employ centralized propulsion systems, which limit fault tolerance, posture control, and robustness under asymmetric flow conditions. Distributed actuation, in contrast, enables decentralized thrust and lift generation, providing redundancy and improved stability in unsteady environments. The spider-inspired hexapod configuration adopted in this work naturally supports such distributed propulsion through bilateral integration of flapping fins within a segmented body, facilitating load redistribution and body-level stabilization. Building on prior structural validation, the present study focuses on evaluating the hydrodynamic performance and propulsion efficiency of this configuration.

The novelty of this work lies in the integration of a six-legged, spider-inspired underwater robot with bilateral flapping fins embedded between leg triads, enabling distributed thrust generation and lift-induced stabilization within a multi-bodied architecture. Unlike conventional underwater robots that rely on centralized or monolithic fin actuation, the proposed configuration decentralizes propulsion across the body, allowing coordinated multi-fin actuation to contribute simultaneously to thrust and stability. In addition, the study adopts a three-level validation framework comprising quasi-steady theoretical force estimation, transient CFD simulations resolving unsteady flow and wake dynamics, and comparative evaluation across multiple flapping regimes. This framework enables systematic assessment of hydrodynamic performance over energy-efficient and high-thrust operating conditions, with specific emphasis on lift-to-power scaling, unsteady efficiency, and wake modulation. The inclusion of full-body flow visualization further allows examination of distributed propulsion effects and wake interactions at the system level, which remain limited in existing fin-based underwater robotic studies.

This paper presents a rigorous, multi-layer analysis of a spider-robot-inspired flapping-fin system with six legs and dual side fins. The study is structured into three main components:

- Theoretical modelling: Lift and drag forces are estimated using classical hydrodynamic equations, Reynolds number analysis, and performance metrics (e.g., lift-to-power ratios).
- CFD simulations: Three-dimensional, transient ANSYS Fluent simulations are performed to obtain pressure contours, velocity wake structures, turbulent kinetic energy, and hydrodynamic force coefficients (lift, drag, and Cd).
- Experimental validation and comparative analysis: A prototype is fabricated and tested in controlled water-tank experiments, and the measured hydrodynamic performance is quantitatively compared with theoretical predictions and CFD results to validate numerical accuracy and unsteady flow effects.

Accordingly, the objective of this study is not to present a finalized field-ready underwater robot, but rather to experimentally and numerically validate the hydrodynamic feasibility of distributed flapping-fin propulsion integrated with a multi-legged body. The prototype serves as a proof-of-concept platform to investigate lift generation, wake characteristics, and energy efficiency prior to full system-level waterproofing and mission-oriented deployment.

## Spider-inspired underwater robot design and fin mechanism

**Fig. 1:**
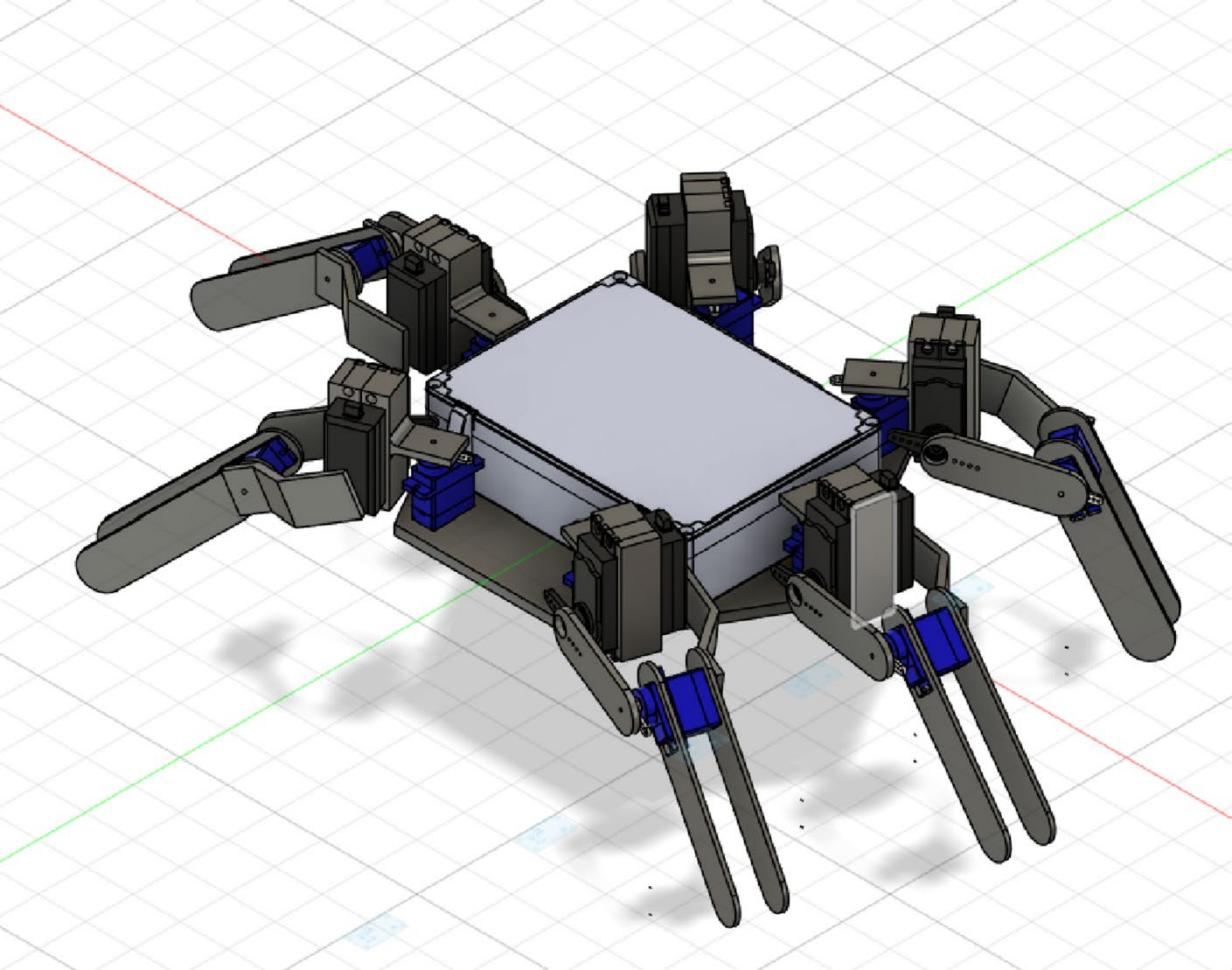
CAD model of the underwater spider robot showing six-legged configuration and central body frame.

**Fig. 2:**
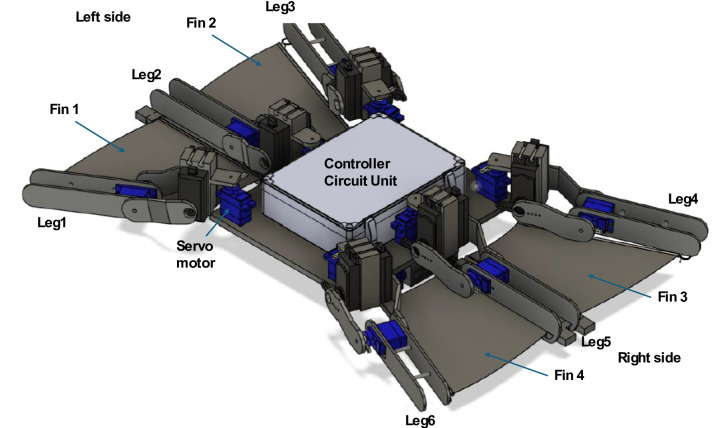
CAD model of the underwater spider robot with fins and its parts.

The underwater spider robot is designed as a hexapod structure comprising six legs symmetrically distributed along the central body. The design of the six-legged underwater spider robot is shown in Fig. 1, where Fig. 2 is with its fins. Each leg is actuated with three degrees of freedom, facilitating ground mobility and underwater stability. The robot’s total weight is approximately 1.2 kg, and buoyancy is managed through neutral floatation techniques and distributed body mass. To facilitate underwater propulsion and lift, two flexible fins are mounted between the three legs on either side of the robot, forming a bilateral propulsion mechanism. The fin material is composed of thermoplastic polyurethane (TPU), chosen for its flexibility and water resistance, while the main body frame utilizes lightweight ABS plastic reinforced with stainless steel linkages for structural integrity. The mechanical layout and symmetric six-legged configuration are adapted from prior gait studies on terrestrial and amphibious robots from Singh et al.^23^, where optimal joint coordination and leg spacing were explored for stability and mobility.

The placement of the servo motors doesn’t differs between the configurations with and without side fins to accommodate the fin integration and associated actuation mechanisms. This modification is purely a packaging adjustment and does not alter the leg kinematic structure, joint degrees of freedom, or actuation sequence. The leg link lengths, joint axes, and control logic remain unchanged between configurations. As a result, the relocation of servos does not affect leg stability or terrestrial locomotion performance, and the robot retains identical gait characteristics in both configurations.

### Side fin configuration and flapping actuation

**Fig. 3:**
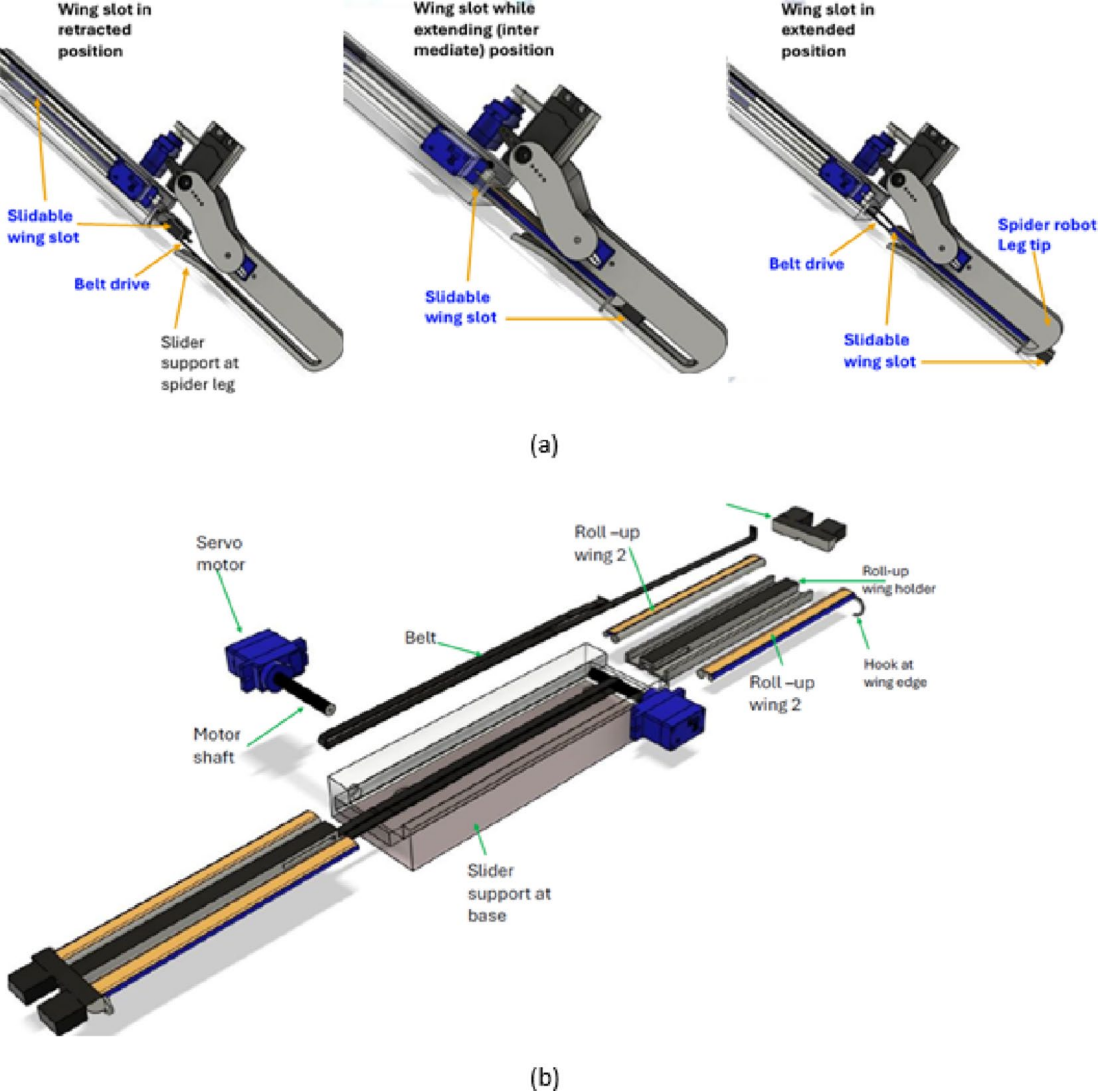
(**a**) Actuation of side fins between legs for distributed flapping propulsion. (**b**) Isometric view of wing deployment through hook-based pulling.

Each side fin is positioned between the front, middle, and rear legs on both the left and right sides. Each flexible side fin, mounted between leg triads, operates as shown in Fig. 3a. These fins are designed to flap vertically, emulating the pectoral fin motions seen in aquatic species such as rays and bony fishes. The flapping is achieved using a geared motor system transmitting rotational motion into oscillatory fin displacement. The typical range of motion for each fin varies between ± 30° to ± 45°, with flapping speeds ranging from 90 to 300 RPM, depending on the operating mode. The structural geometry of each fin has a span of approximately 0.20 m and a chord length of 0.12 m, with a total surface area of 0.026 m^2^. These dimensions were selected to provide sufficient lift force to contribute to partial or complete buoyant lift.

The slidable wing slot incorporated at the fin root, as shown in Fig. 3a, serves a dual mechanical function. First, it guides the translational motion of the fin during deployment, ensuring smooth and repeatable extension from the folded to the operational position. Second, it constrains the fin motion within a predefined path, preventing lateral misalignment and excessive deformation during flapping. This guided-slot mechanism enhances deployment reliability while maintaining compact stowage during terrestrial operation.

From Fig. 3b, the mechanism ensures compact stowage of the fins during terrestrial operation and enables rapid transition to flapping propulsion in water. The simplicity of the hook-actuation design enhances mechanical reliability under submerged conditions, allowing the robot to switch between land and underwater modes efficiently. This adaptability makes the configuration particularly suited for amphibious robots requiring modularity and space-saving deployment. Arrows indicate fin flapping direction and dashed outlines represent the angular limits (± 30°–±45°).

Figure 3b illustrates the detailed design of deployment configuration of the side fin using a hook-actuation system, which enables compact stowage and quick transition into propulsion mode. It presents an isometric view of the wing deployment mechanism using a hook-based pulling system. This configuration is designed to enable passive or semi-active deployment of the side fins during underwater operations. The hook system ensures that the fin remains securely folded during ground mobility or standby conditions and can be rapidly engaged into the flapping position when transitioning to aquatic locomotion. This mechanism not only enhances the robot’s adaptability in multi-environment settings but also contributes to streamlined storage and transport configurations. Such deployable fin systems are particularly useful in amphibious or modular underwater robots, where spatial constraints and reconfigurability are critical. While similar approaches have been used in foldable wing or arm designs, this implementation emphasizes mechanical simplicity and reliability under submerged conditions.

### Operational objectives and fluid interaction

The dual flapping fins are primarily responsible for generating lift and thrust during underwater locomotion. By tuning the flapping frequency and amplitude, the robot can either hover in place, move forward, or generate upward lift to counteract gravity. Left and right fin coordination is used to stabilize roll and yaw during manoeuvring. The overall control involves synchronizing the two side fins for symmetrical propulsion or introducing phase differences for turning and dynamic balance. This mechanism enables biologically inspired, energy-efficient swimming with significant adaptability to flow disturbances and terrain undulations.

To ensure the validity and robustness of the proposed underwater spider robot design, a three-stage evaluation framework was adopted. This included:

1. Theoretical calculations based on fluid dynamics principles.
2. Computational Fluid Dynamics (CFD) simulations using ANSYS Fluent.
3. A comparative analysis between analytical and simulated outcomes.

This structured methodology allows both predictive estimation and empirical confirmation of hydrodynamic lift and drag forces, as well as associated flow behaviours around the flapping fins.

### Control electronics and power architecture

**Fig. 4:**
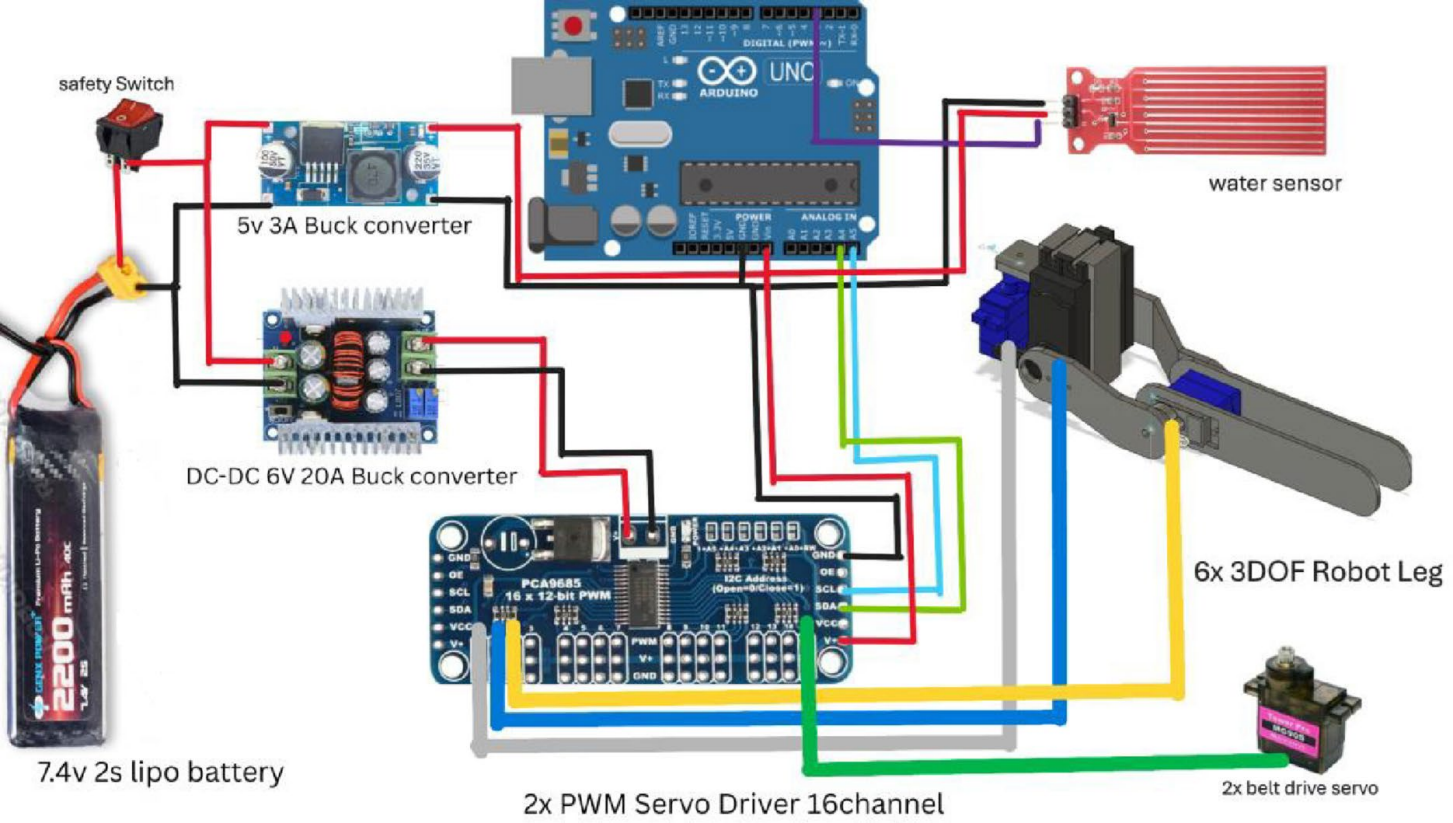
Control electronics and power distribution system of the underwater spider robot, showing integration of Arduino Uno, PCA9685 PWM drivers, dual buck converters, Li-Po battery, and water sensor for amphibious operation.

To enable coordinated actuation of the six-legged underwater spider robot and the bilateral flapping fins, a compact electronic control system was developed (Fig. 4). The architecture is based on an Arduino Uno microcontroller interfaced with two PCA9685 16-channel PWM servo drivers for controlling up to 32 servo channels, which are required for the 6 × 3 DOF leg joints and dual fin actuation.

The control logic for amphibious operation was implemented in Arduino and is summarized in pseudocode at Appendix 2. i.e. the pseudocode outlines the autonomous control logic implemented on the Arduino microcontroller for amphibious operation of the spider-inspired robot. It begins with initialization of servo channels through PCA9685 drivers and configuration of the water sensor. During execution, the system continuously monitors the water sensor to determine the environment. In dry conditions, a tripod gait function is activated, coordinating alternate sets of three legs to achieve stable walking. When water is detected, the robot switches to a fin-flapping mode, where bilateral fins are actuated synchronously for forward swimming. Turning manoeuvres are achieved by introducing phase offsets between left and right fins. This abstraction highlights how environmental sensing governs locomotion mode, ensuring smooth transitions between land and underwater propulsion.

A dual buck converter setup was employed to regulate power from a 7.4 V, 2200 mAh 2 S Li-Po battery: a 5 V/3 A converter powers the Arduino and peripheral sensors, while a 6 V/20 A converter provides sufficient current for the high-torque servomotors. A safety switch is integrated between the battery and converters for reliable shutdown. To incorporate autonomous reactive behaviour, a water immersion sensor is interfaced with the Arduino. The sensor enables automatic detection of aquatic conditions, triggering mode transitions between terrestrial walking and underwater fin-based swimming. Control commands are relayed through the PWM drivers, ensuring synchronized tripod gait on land and bilateral flapping propulsion in water. This modular hardware framework ensures lightweight operation, sufficient current delivery for simultaneous leg and fin actuation, and a reliable switching mechanism between amphibious locomotion modes. The selection of a 7.4 V, 2200 mAh (2 S) Li-Po battery is intended to support short-duration, proof-of-concept experiments conducted in a controlled water-tank environment. The present study focuses on validating the hydrodynamic feasibility and coordinated actuation of distributed flapping fins rather than long-duration endurance operation. Continuous or extended underwater deployment involving simultaneous actuation of multiple servomotors would require higher-capacity energy storage, optimized power management, and efficiency-oriented control strategies, which are identified as important directions for future work.

The present prototype was developed as a laboratory-scale hydrodynamic validation platform rather than a long-duration field-deployable underwater system. Accordingly, commercial off-the-shelf electronics (Arduino Uno, servos, and Li-Po battery) were used with short-duration water exposure under controlled tank conditions. Full waterproofing solutions such as IP-rated enclosures, conformal coatings, sealed connectors, and pressure-compensated housings are part of planned future iterations. The water immersion sensor was employed solely for binary environment detection during short-duration tests, and signal integrity was maintained through insulated wiring and controlled exposure conditions.

## Theoretical modelling of flapping fin hydrodynamics

To establish a foundational understanding of the hydrodynamic forces acting on the flapping fins, a quasi-steady theoretical approach is adopted. The following assumptions are made:

- The fluid is incompressible and Newtonian (ρ = 1000 kg/m^3^, µ = 0.001 Pa s).
- The fin undergoes sinusoidal flapping motion at a steady amplitude and frequency.
- Flow separation and cavitation are negligible within the operational Reynolds number range (Re ≈ 10^4^ to 10^5^).
- The fin behaves like a hydrofoil, and its motion induces unsteady lift and drag due to added mass and wake interactions.

In the numerical model, the legs of the spider robot were treated as static bodies to isolate the hydrodynamic contribution of the flapping fins. This simplification allows focused evaluation of fin-generated lift and wake behavior while avoiding confounding effects from leg-induced drag or auxiliary thrust. Although leg–fluid interactions may influence total resistance during free swimming, their exclusion does not affect the primary objective of this study, which is comparative validation of distributed flapping-fin propulsion. The fin tip velocity reported in this study represents a kinematic input parameter rather than the actual swimming speed of the robot. Conversion from fin-induced thrust to steady swimming speed requires a full thrust–resistance equilibrium analysis, including body and leg drag.

### Lift and drag force estimation

A fixed body-centered Cartesian coordinate system is adopted to define hydrodynamic forces acting on the flapping fin. The streamwise $\:\mathrm{x}$-direction corresponds to the nominal forward swimming direction of the robot, the vertical $\:\mathrm{z}$-direction represents the lift direction normal to the mean plane of motion, and the $\:\mathrm{y}$-direction completes the right-handed coordinate system. Accordingly, lift is defined as the force component acting along the $\:\mathrm{z}$-axis, while thrust (or drag) corresponds to the force component acting along the $\:\mathrm{x}$-axis.

In the present theoretical formulation, mechanical input power is estimated based on the drag-related resistance encountered by the fin during oscillatory motion. This approximation is commonly used in bio-inspired flapping propulsion studies, where actuator power is primarily expended in overcoming hydrodynamic resistance associated with fin translation through the fluid. Although lift generation contributes to useful force output, it does not directly represent resistive power loss and is therefore not included in the mechanical power estimation. The drag force considered in the power calculation corresponds to the component opposing the instantaneous fin motion direction. This simplified representation provides a conservative estimate of actuator power requirements and enables consistent comparison of lift-to-power performance across different flapping conditions.

The hydrodynamic lift (L) and drag (D) forces are estimated using classical fluid dynamics equations:

$${\rm{L}} = {\rm{0}}{\rm{.5}} \times \rho \times {\rm{A}} \times {\rm{C\_L:}}{{\rm{V}}^2}$$

$${\rm{D}} = {\rm{0}}{\rm{.5}} \times \rho \times {\rm{A}} \times {\rm{C\_D:}}{{\rm{V}}^2}$$

where:

- ρ = 1000 kg/m^3^ (density of water).
- A = 0.026 m^2^ (fin surface area).
- C_L = 1.4 (lift coefficient for flexible flapping fins).
- C_D = 0.28 (drag coefficient, averaged from simulations).
- V = fin tip velocity (m/s), varies between 0.6 m/s and 2.0 m/s.

The lift and drag coefficients used in the theoretical analysis ($\:{\mathrm{C}}_{\mathrm{L}}=1.4$ and $\:{\mathrm{C}}_{\mathrm{D}}=0.28$) were selected based on reported ranges for flexible flapping fins and hydrofoils operating at comparable Reynolds numbers in prior bio-inspired propulsion studies. Previous investigations on oscillating and flexible fins have shown that unsteady vortex formation and effective camber enhancement can result in lift coefficients exceeding those of rigid hydrofoils, particularly during mid-stroke phases. The chosen coefficients therefore represent representative, time-averaged values intended to provide a conservative baseline for theoretical force estimation rather than precise instantaneous predictions. These coefficients were further cross-checked against preliminary CFD results, which showed that the resulting theoretical lift and drag trends remain consistent with numerically obtained force magnitudes, especially within the moderate flapping velocity regime.

### Reynolds number estimation

In this study, the characteristic velocity U used in the Reynolds number definition is taken as the flapping fin tip velocity rather than a prescribed incoming flow velocity. This choice is intentional and appropriate for self-propelled, bio-inspired flapping systems operating in quiescent water, where thrust and flow are generated primarily by fin motion rather than by an externally imposed freestream. For such oscillatory propulsion mechanisms, the fin tip velocity represents the dominant relative velocity between the structure and the surrounding fluid and is therefore commonly adopted in Reynolds number estimation for flapping foils and swimming robots. This approach has been widely used in studies of flapping hydrofoils, flexible fins, and fish-like propulsion, particularly under still-water or self-propelled conditions, where no uniform incoming flow exists.

Reynolds number is used to characterize the flow regime around the fin:

$${\rm{Re = (}}\rho \times {\rm{V}} \times {\rm{L}})/\mu$$

where:

- L = 0.12 m (chord length of fin).
- µ = 0.001 Pa·s (dynamic viscosity of water).
- V = flapping fin tip velocity.

This equation helps evaluate flow transition and vortex dynamics around the fin during flapping.

where V is the peak flapping tip velocity, ρ is the fluid density, µ is the dynamic viscosity of water and L is the characteristic chord length of the fin. In this work, the characteristic velocity used in the Reynolds number definition is taken as the flapping fin tip velocity rather than an imposed incoming flow velocity. This choice is intentional and appropriate for self-propelled flapping systems operating in quiescent or still-water conditions, where no uniform freestream exists and the dominant relative motion between the structure and fluid is induced by fin kinematics. Similar formulations have been widely adopted in studies of flapping hydrofoils and swimming robots under self-generated flow conditions.

The fin tip velocity was determined directly from the prescribed flapping kinematics, defined by the fin span, flapping amplitude, and actuation frequency. The selected velocity range (0.6–2.0 m/s) corresponds to flapping frequencies between 90 and 300 RPM, which are achievable with the selected actuators and remain within biologically relevant Strouhal number regimes. Lower velocities (0.6–0.8 m/s) were chosen to represent energy-efficient swimming and hovering modes, while higher velocities were included to examine high-thrust or burst-operation conditions. The same fin tip velocity range was consistently used in theoretical calculations, CFD simulations, and experimental trials to ensure coherence across the three-tier validation framework.

### Power input and performance efficiency

The mechanical power required to overcome hydrodynamic drag at the fin is estimated using:

$${\rm{P}} = {\rm{D}} \times {\rm{V }}$$

The performance efficiency of the flapping mechanism is then evaluated using a lift-to-power ratio:

$$\eta = {\rm{L / P}}{\rm{.}}$$

This ratio indicates the thrust generation capability per unit of energy input. It is a critical parameter in bio-inspired propulsion design, helping identify energy-optimal flapping conditions.

### Unsteady effects and bio-inspired scaling considerations

While the above formulations assume a quasi-steady state, it is important to acknowledge the presence of unsteady phenomena such as added mass effects, vortex lift, and wake interactions, especially prominent at moderate-to-high Reynolds numbers (10^4^–10^5^). These unsteady contributions, though not directly captured in static analytical models, influence the instantaneous lift and drag forces during fin reversal and stroke transitions. Studies on flapping hydrofoils and flexible fins have shown that leading-edge vortex formation and delayed stall can temporarily enhance lift^3,10^.

To better understand the efficiency and bio-inspired fidelity of the system, the Strouhal number (St) is used as a non-dimensional indicator of propulsive effectiveness. Biological swimmers such as fish and rays typically operate within an optimal Strouhal range of 0.2–0.4, balancing thrust generation with energy efficiency^3,11^. Although not computed in this section, later simulation results confirm that the proposed fin design aligns with this efficient regime.

The analytical formulation adopted in this study is based on a quasi-steady assumption, wherein instantaneous hydrodynamic forces are estimated using time-averaged lift and drag coefficients. This approach is intentionally employed to provide a simplified baseline for force estimation and parameter scaling, and it has been widely used in preliminary analysis of flapping foils and bio-inspired propulsion systems. While this formulation does not explicitly account for unsteady contributions such as added-mass effects, vortex-induced lift, and wake interaction during stroke reversal, these mechanisms are expected to enhance instantaneous force production, particularly at moderate-to-high Reynolds numbers. As a result, deviations between quasi-steady predictions and CFD or experimental results are anticipated, especially at higher flapping velocities where unsteady effects dominate. In the present work, the quasi-steady model is therefore used primarily for trend analysis and order-of-magnitude estimation, while detailed unsteady hydrodynamic behaviour and cycle-averaged forces are captured through transient CFD simulations and validated experimentally.

Additionally, while constant coefficients C_L_ and C_D_ are used for theoretical estimation, these values may vary dynamically due to instantaneous flow separation and pressure redistribution. Therefore, the theoretical results serve as a baseline framework, which is subsequently refined through transient CFD simulations incorporating real-time fluid–structure interactions.

## CFD simulations and hydrodynamic validation

Computational Fluid Dynamics (CFD) simulations were performed using ANSYS Fluent 2025 R1 to evaluate the fluid–structure interaction and hydrodynamic behaviour of the flapping fins. The imported CAD model of a single side fin assembly was enclosed in a fluid domain, representing still water. A structured mesh with boundary layer refinement was applied to resolve near-wall flow behaviour and minimize numerical diffusion. The pressure-based solver was employed with double-precision accuracy. A steady-state formulation was initially used to estimate average flow quantities, followed by unsteady simulations for detailed wake dynamics. The standard k–ε turbulence model with enhanced wall treatment was adopted for all simulations.

The standard k–ε turbulence model with enhanced wall treatment was selected in this study due to its numerical robustness, computational efficiency, and proven reliability for time-averaged force prediction in oscillatory and bio-inspired propulsion problems. While more advanced models such as k–ω SST can provide improved near-wall and separation resolution, the k–ε model offers stable convergence for transient simulations involving complex moving boundaries. The limitations of k–ε in capturing fine-scale near-wall vortical structures are acknowledged and are considered in the interpretation of the CFD results.

### Boundary conditions and solver parameters

The computational domain represents a quiescent-water environment, with the inlet velocity specified to match the characteristic fin tip velocity used for non-dimensional scaling and Reynolds number consistency (ranging from 0.6 to 2.0 m/s). This inlet condition does not represent a steady swimming speed, but rather provides a reference velocity for resolving relative fin–fluid motion in the numerical framework. The fin surface is modelled as a moving wall with prescribed time-dependent flapping kinematics, making the simulation inherently transient and dominated by oscillatory motion rather than steady inflow. A pressure-outlet boundary condition (0 Pa gauge pressure) is applied at the outlet, and no-slip conditions are enforced on all solid surfaces. Gravity is neglected, as the focus is on hydrodynamic force generation due to fin motion. Time steps are selected to resolve the flapping frequency (90–300 RPM), and simulations are advanced over multiple complete flapping cycles until periodic steady-state behaviour is achieved. Hydrodynamic forces are extracted as time histories of lift and drag, and cycle-averaged values are computed from the final converged cycles. Residuals for continuity, momentum, and turbulence quantities are monitored and maintained below $\:{10}^{-5}$.

**Fig. 5:**
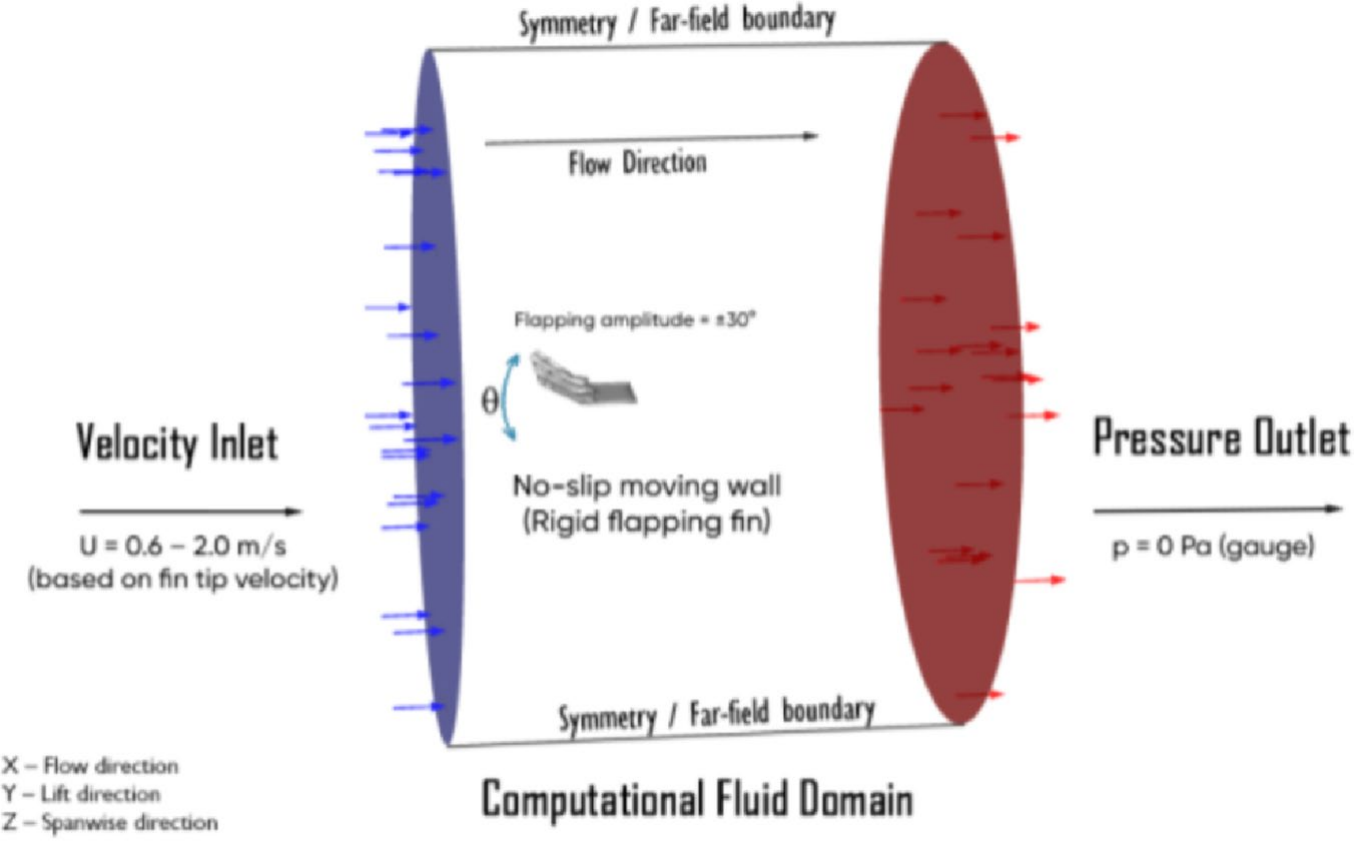
Schematic representation of the computational fluid domain and boundary conditions used for the flapping-fin CFD simulations.

A schematic representation of the numerical simulation setup is shown in Fig. 5. The computational domain is defined as a three-dimensional cylindrical fluid volume to minimize boundary interference effects. The flapping fin is placed at the centre of the domain. A uniform velocity inlet is prescribed at the upstream boundary, with inlet velocity ranging from 0.6 to 2.0 m/s based on the fin tip velocity. A pressure outlet condition with zero-gauge pressure is applied at the downstream boundary. The lateral surfaces of the domain are treated as symmetry/far-field boundaries. The upper and lower computational boundaries were also modelled using symmetry/far-field boundary conditions rather than no-slip wall boundaries. These boundaries were positioned sufficiently far from the flapping fin to minimize artificial confinement effects and to approximate an effectively unbounded quiescent-water environment. Consequently, the low-velocity regions visible near the domain boundaries in some contour plots correspond to undisturbed ambient fluid zones resulting from flow decay and contour scaling, rather than physically imposed zero-velocity wall conditions. This treatment ensures that the computed wake evolution and hydrodynamic forces are governed primarily by fin kinematics rather than numerical boundary interference. The fin surface is modelled as a no-slip moving wall undergoing prescribed flapping motion with an amplitude of ± 30°. This setup allows isolation of the hydrodynamic forces generated by fin kinematics while maintaining numerical stability and consistency across different flapping conditions.

#### Grid independence and transient validation

To ensure numerical reliability, both spatial mesh independence and temporal convergence were assessed for the flapping fin simulation. Three grid densities were tested: coarse (2.5 million cells), medium (4.8 million cells), and fine (8.1 million cells). Inflation layers were applied near the fin surfaces, ensuring that the first-layer y⁺ remained < 1 for accurate near-wall turbulence resolution. The pressure and velocity gradients around the fin were observed to converge with the medium and fine meshes.

**Table 1:** Mesh independence study results at U = 0.73 m/s.

Grid level	Elements	C_L_	C_D_	% Error in C_L_
Coarse	2.5 million	1.212	0.856	6.0%
Medium	4.8 million	1.268	0.878	1.9%
Fine	8.1 million	1.292	0.882	–

Table 1, Shows C_L_ and C_D_ for coarse, medium, and fine meshes and the percentage error in C_L_ relative to the finest grid. The medium mesh (4.8 million cells) was selected for final simulations due to its balance between accuracy and computational cost.

#### Transient convergence and time-step sensitivity

Transient simulations were performed for six complete flapping cycles using a fixed time step of Δt = 0.001 s. The force–time histories of lift and drag coefficients demonstrated that a periodic steady state was reached after approximately three flapping cycles. Accordingly, only the final three cycles were used for extracting time-averaged hydrodynamic metrics. A time-step sensitivity analysis conducted for Δt = 0.0005 s, 0.001 s, and 0.002 s resulted in variations of less than 2.5% in the lift coefficient (C_L_), validating the selected time step for all unsteady simulations. To further ensure the accurate acquisition of transient hydrodynamic information and to support grid selection, a time-history-based grid independence study was conducted. Three mesh densities coarse (≈ 2.5 million cells), medium (≈ 4.8 million cells), and fine (≈ 8.1 million cells) were evaluated under identical flapping kinematics and boundary conditions. For each grid, instantaneous lift and drag coefficients were monitored over multiple flapping cycles.

The time-history curves indicate that, while the coarse mesh exhibits reduced force amplitude and minor phase lag, the medium and fine meshes produce nearly identical periodic waveforms once the solution reaches steady oscillation. The peak-to-peak variation in lift coefficient between the medium and fine grids was less than 2%, confirming negligible grid influence on transient force evolution. Based on this agreement and computational efficiency considerations, the medium grid was selected for all subsequent unsteady simulations. To further demonstrate transient accuracy, time-history curves of lift and drag coefficients for coarse, medium, and fine meshes are presented. These curves show that while the coarse mesh exhibits reduced force amplitude and minor phase lag, the medium and fine meshes produce nearly identical periodic responses, confirming grid-independent transient force prediction.

### Simulation results and force extraction

The simulated pressure fields showed gradual development of lift-generating pressure differential across the fin surface. Lift and drag forces were extracted using surface integral monitors over the fin boundary. Contours of static pressure, velocity streamlines, and turbulent kinetic energy (TKE) were used to visualize vortex formation and flow recirculation zones.

The lift force increased with tip velocity, aligning closely with theoretical estimations. For example, at 0.73 m/s, the simulated lift was approximately 5.3 N compared to 9.7 N analytically. At 2.0 m/s, the lift reached 40.2 N (simulation), affirming the model’s nonlinear lift–velocity relationship. A mesh convergence study was conducted to confirm that the CFD results are independent of grid density. The lift and drag coefficients for three mesh sizes are reported in Table 1. The medium mesh (4.8 million cells) produced results within 2% of the fine mesh, confirming its adequacy for all subsequent simulations.

In this study, the characteristic velocity is derived from the prescribed fin flapping kinematics and corresponds to the fin tip velocity. This velocity is used solely as a kinematic scaling parameter for Reynolds number estimation and numerical reference in the CFD simulations. It does not represent the actual self-propelled swimming speed of the robot, which would require a full thrust–drag equilibrium analysis of the body–fin system.

#### Static pressure distribution on flapping side fin

To analyse the hydrodynamic behaviour and thrust-generating capability of the side fins of the underwater spider robot, Computational Fluid Dynamics (CFD) simulations were performed using ANSYS Fluent 2025 R1. A single side-wing configuration (comprising two fin surfaces between three legs) was isolated for the simulation under representative flapping conditions. The wing flapping motion was modelled at a tip velocity of 0.73 m/s and a rotational speed of 110 RPM, replicating the operating regime used in underwater mobility trials. The objective was to verify whether the generated pressure forces were sufficient to support lifting and propulsion functions.

**Fig. 6:**
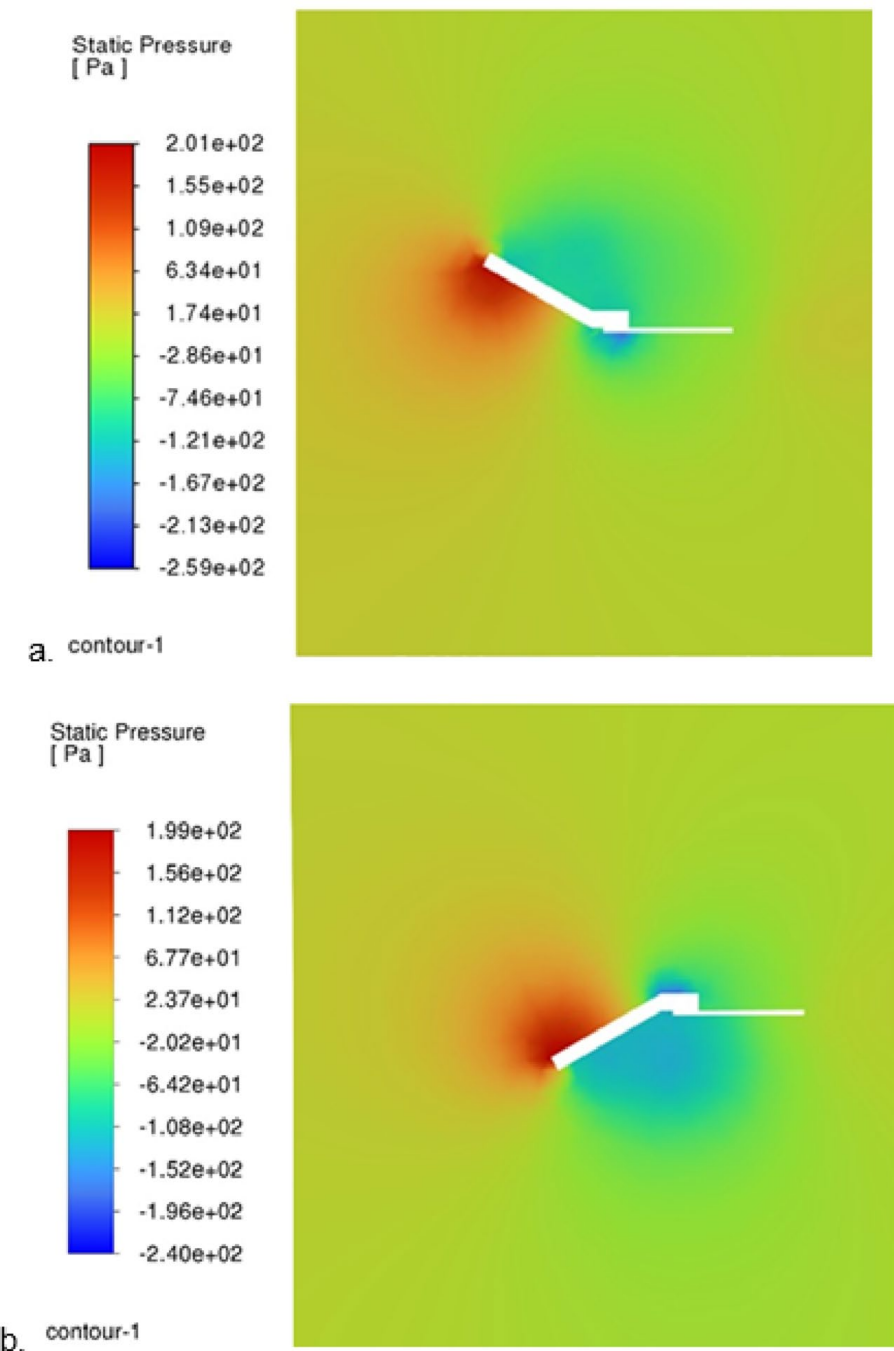
(**a**) Static pressure contour around side wing during flapping, and (**b**) Pressure distribution over side wing under varied orientation.

Figure 6a shows the static pressure contour of the one-sided wing structure during active flapping. A well-defined pressure differential is observed across the fin surface. The leading surface exhibits a high-pressure region, reaching up to + 201 Pa, shown in red, while the trailing surface shows a low-pressure zone, down to − 259 Pa, shown in blue. This pressure gradient induces an effective upward lift force on the fin. The simulation estimates a lift force of approximately 5.3 N, which exceeds the target requirement of 4.7 N per side to lift half of the 1.2 kg robot body (total weight ≈ 11.77 N). This confirms that the fin design is capable of contributing significantly to vertical lift and manoeuvrability.

Figure 6b presents a second simulation case where the wing geometry or angle was slightly modified, simulating the dynamic asymmetry introduced during a real flapping stroke. The pressure field shows a similarly strong distribution, with the leading surface reaching + 199 Pa and the trailing surface dropping to − 240 Pa. Despite the subtle shift in geometry or angular offset, the pressure differential remains adequate to produce over 5 N of lift, validating the robustness of the fin design under varying flapping angles. These results confirm that both fins—when actuated in sync or in phase opposition—can generate sufficient propulsion and stabilize the robot in underwater environments. These simulations emphasize the aerodynamic (hydrodynamic) efficiency of the fin design. The observed pressure contours reflect optimized flow behavior across the fin surface, with minimal stagnation and strong boundary interaction, ideal for thrust and lift in underwater robotic applications.

#### Flow behavior analysis around side wing

To evaluate the fluid dynamic performance of the spider robot’s side-mounted flapping fin, further post-processing of the ANSYS Fluent simulation results was carried out. This includes analysis of the Turbulent Kinetic Energy (TKE) and Velocity Magnitude distributions in the surrounding fluid domain. These analyses provide insight into the wake characteristics, energy transfer mechanisms, and propulsion efficiency of the fin design.

**Fig. 7:**
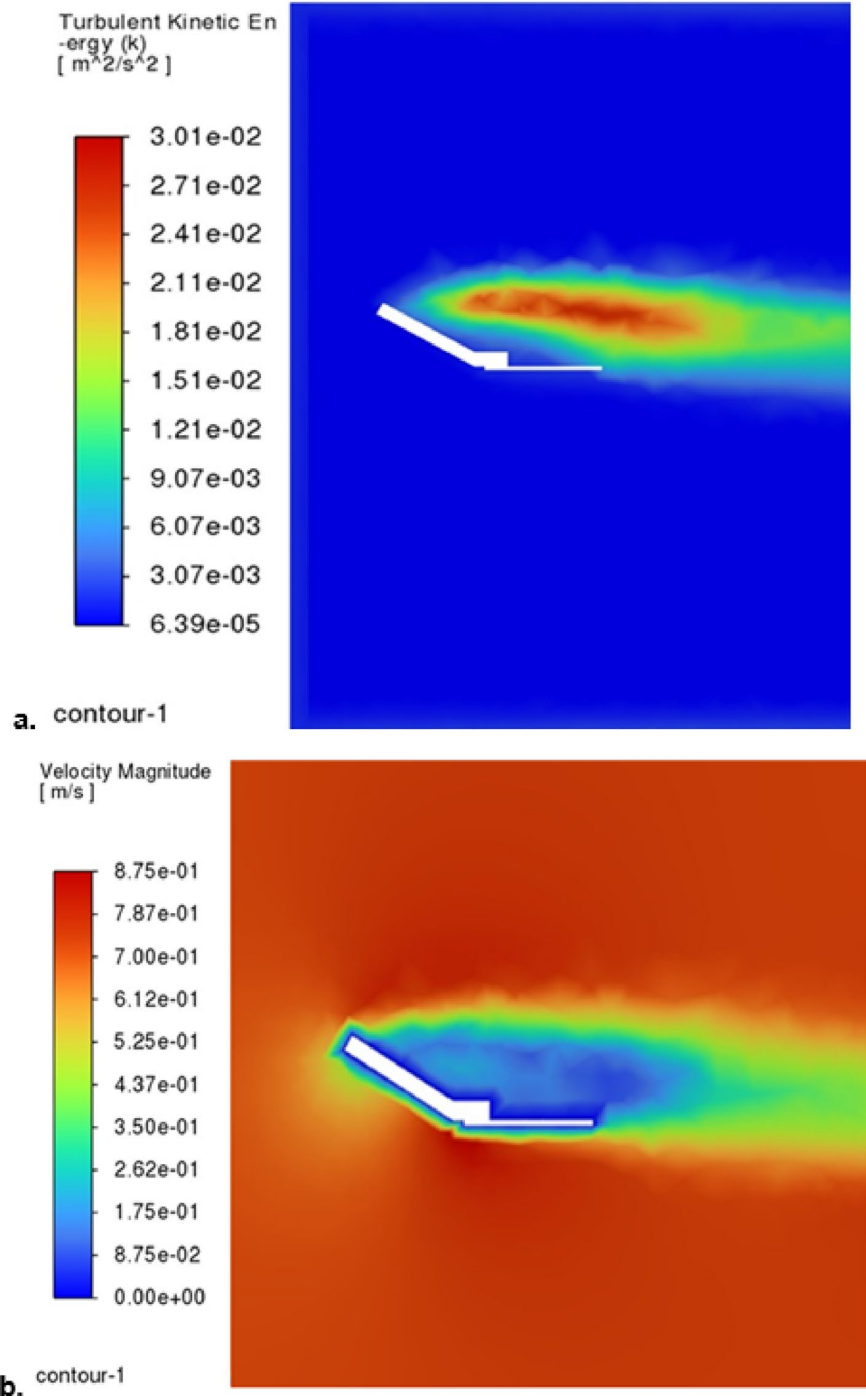
(**a**) Turbulent kinetic energy contour around the side wing, and (**b**) Velocity magnitude contour around the side wing.

Figure 7a illustrates the turbulent kinetic energy contour around the side wing. The region downstream of the fin shows a distinct wake structure with elevated TKE levels, reaching up to 0.03 m^2^/s^2^, indicating the presence of vortex shedding and flow disturbance due to the flapping motion. The central wake zone appears concentrated and elongated, indicating that the turbulent energy is confined and directional, a behavior aligned with efficient thrust generation. The turbulence originates primarily near the leading and trailing edges of the fin, suggesting that the motion-induced boundary layer separation and vortex formation are controlled and symmetrical. Such characteristics are beneficial for underwater propulsion, as they prevent erratic flow-induced vibrations and ensure smooth locomotion.

Figure 7b displays the velocity magnitude distribution across the domain. A low-velocity wake is clearly observed trailing behind the fin, surrounded by high-velocity gradients, especially near the fin tip. The simulation confirms that the flapping tip velocity of 0.73 m/s results in flow speeds exceeding 0.8 m/s around the outer zones, consistent with intended input conditions. The velocity deficit behind the fin reflects the energy imparted to the water by the flapping action, forming the basis of thrust generation. Importantly, the flow remains largely unidirectional and symmetrical, indicating that the fin does not introduce flow instabilities or lateral disturbances. This supports stable forward motion and controllability of the robot in an underwater environment. These two figures together, confirm that the wing structure and flapping configuration not only generate sufficient pressure for lift (as discussed in Sect.  2.4.1) but also create a coherent wake that supports efficient underwater movement. The combination of moderate turbulence and streamlined velocity patterns validates the suitability of the wing design for dynamic underwater applications. The confined turbulent kinetic energy distribution further indicates that the wake remains coherently organized rather than chaotically separated, which is beneficial for maintaining stable momentum transfer and minimizing unsteady lateral force fluctuations during propulsion.

#### Force coefficients and lift-drag performance

**Fig. 8:**
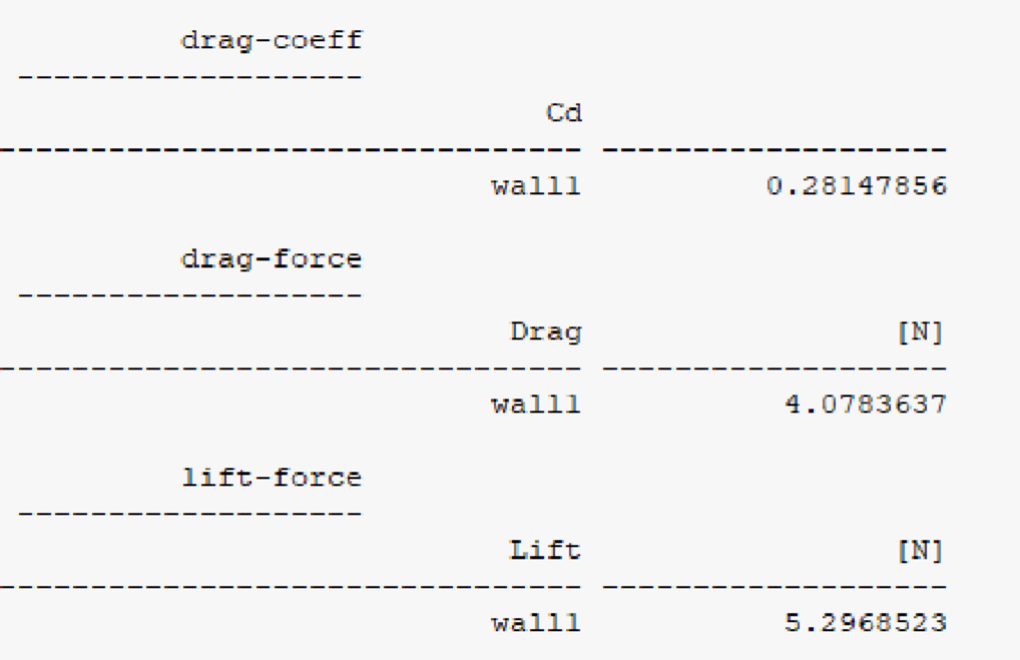
CFD-computed hydrodynamic performance metrics for one side wing of the underwater spider robot under flapping condition (tip speed: 0.73 m/s, RPM: 110).

To quantify the hydrodynamic efficiency of the flapping side wing, the lift and drag forces acting on the fin were computed from the pressure and velocity distributions in ANSYS Fluent as shown in Fig. 8. These force components are critical for evaluating the thrust-lift trade-off and assessing the wing’s ability to overcome fluid resistance while generating sufficient upward force. The simulation results yielded a lift force of approximately 5.30 N, exceeding the required per-side target of 4.7 N to support the 1.2 kg robot. The corresponding drag force was measured as 4.08 N, resulting in a lift-to-drag ratio (L/D) of approximately 1.30, which is favourable for bioinspired underwater propulsion where balance between vertical lift and forward drag must be maintained. The drag coefficient (Cd) was calculated as 0.281, which indicates moderate fluid resistance. This value suggests that while the fin encounters some drag due to its motion through water, the design remains streamlined enough to prioritize lift generation without excessive energy loss. The force distribution aligns with the pressure and velocity field contours shown earlier (Figs. 5, 6, 7 and 8), further validating the effectiveness of the fin geometry and flapping configuration.

#### Flow evolution at characteristic phases of a flapping cycle

To improve physical interpretation of the flapping-fin hydrodynamics, the flow field is analysed at characteristic instants within a single flapping cycle. These phase-resolved snapshots enable direct correlation between fin kinematics, pressure evolution, vortex formation, and instantaneous force generation. To illustrate the variation laws of the unsteady hydrodynamic response and to improve physical interpretation of the flapping mechanism, the flow field evolution was analysed at characteristic instants within a single flapping cycle. One representative cycle was divided into four key phases based on fin kinematics: (i) start of downstroke, (ii) mid-downstroke, (iii) stroke reversal (bottom dead centre), and (iv) mid-upstroke. These phases capture the dominant changes in fin orientation, velocity, and acceleration that govern force generation. At the start of the downstroke, the fin accelerates from rest, inducing a rapid pressure buildup on the leading surface while the wake remains relatively undeveloped. This acceleration-dominated phase contributes to added-mass effects and initiates vortex formation near the leading edge. During the mid-downstroke, the fin reaches near-maximum translational velocity, producing a strong pressure differential between the pressure and suction sides. A coherent wake structure begins to form downstream, corresponding to peak instantaneous lift and thrust generation.

At the stroke reversal, the fin velocity momentarily approaches zero while its acceleration changes sign. During this phase, the pressure distribution weakens and the previously shed vortices are convected downstream. Although instantaneous lift reduces locally, this phase plays a critical role in wake reorientation and energy transfer to the fluid. During the mid-upstroke, the fin again accelerates, generating an opposing but symmetric pressure distribution. A new set of vortical structures forms with reversed orientation, sustaining periodic force production over the cycle. The phase-resolved analysis reveals that the hydrodynamic forces are not uniformly distributed over the flapping period but are instead dominated by the mid-stroke phases, where fin velocity and pressure gradients are highest. This periodic evolution of pressure, velocity, and wake structure explains the sinusoidal nature of the lift and drag time histories observed in transient simulations and highlights the importance of unsteady effects in flapping-based propulsion.

The phase-resolved analysis demonstrates that hydrodynamic force generation in the distributed flapping-fin system is governed primarily by unsteady vortex dynamics and pressure-gradient evolution throughout the flapping cycle. During the mid-stroke phases, maximum fin velocity produces intensified circulation and strong pressure differentials across the fin surfaces, resulting in peak lift and thrust generation. The formation of coherent leading-edge vortices further enhances instantaneous force production by delaying local flow separation and increasing effective circulation around the fin. In contrast, stroke-reversal phases are dominated by wake reorientation and added-mass effects, during which previously shed vortices are convected downstream before the subsequent acceleration phase begins. These observations confirm that the propulsion mechanism is inherently unsteady and cannot be fully interpreted using purely quasi-steady hydrodynamic assumptions.

### Hydrodynamic performance analysis at reduced flapping velocity (condition 2)

To further examine the impact of reduced flapping speed on the hydrodynamic efficiency of the underwater spider robot’s wing, a secondary simulation was conducted with a fin tip velocity of 0.6 m/s and a flapping rate of 90 RPM. This condition represents a lower-energy mode suitable for energy-saving or manoeuvring operations. The pressure field, velocity profile, turbulence distribution, and resulting force components are evaluated below.

**Fig. 9:**
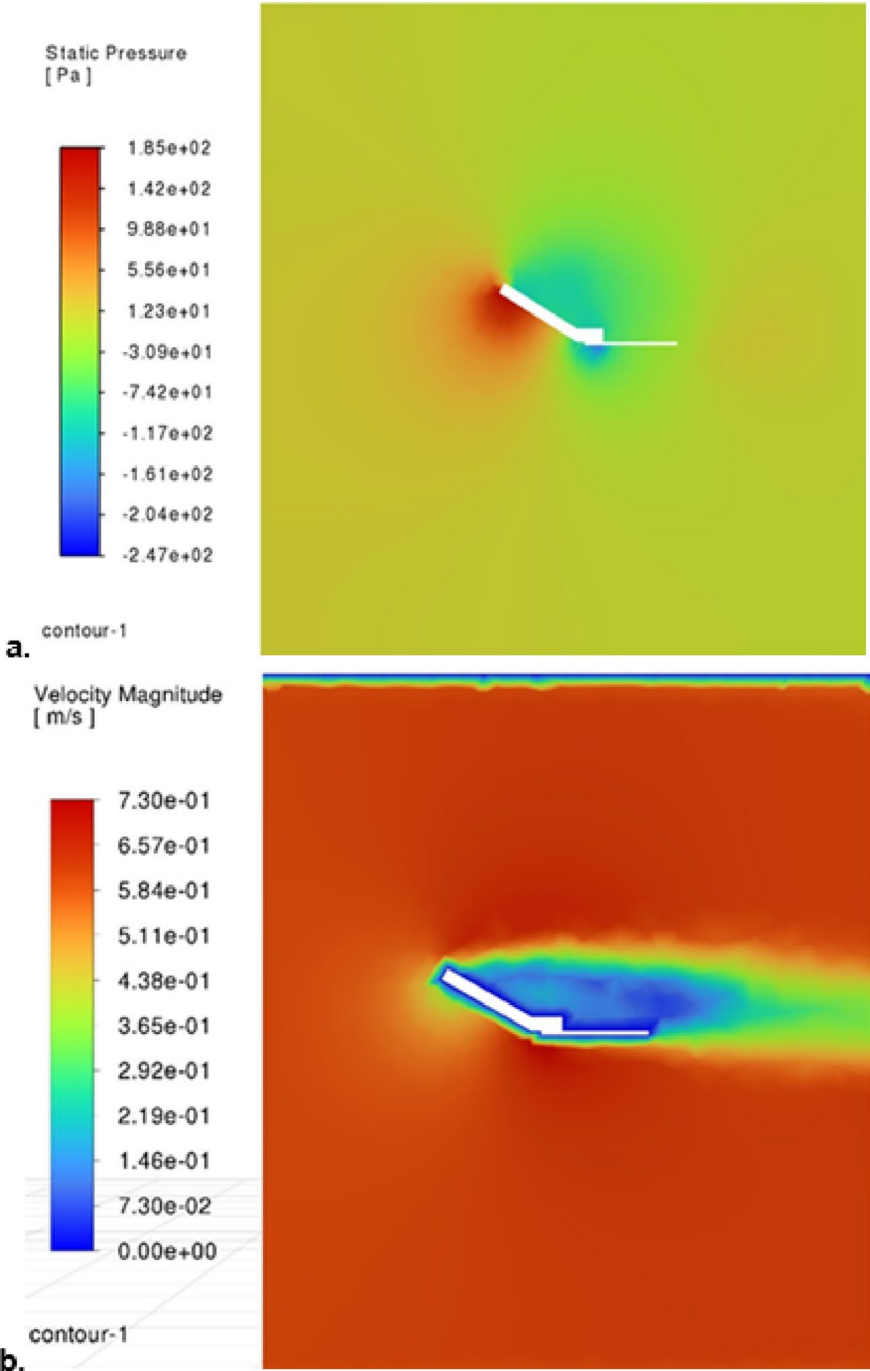
(**a**) Static pressure contour around the side wing at 0.6 m/s tip velocity and 90 RPM, and (**b**) Velocity magnitude contour at reduced flapping condition showing wake profile behind the wing.

Figure 9a shows the static pressure distribution around the side wing during the lower flapping velocity condition. A noticeable pressure differential is observed across the wing surface, with maximum positive pressure reaching approximately 185 Pa on the leading side (in red), and a corresponding negative pressure on the trailing side (in blue). This pressure gradient still produces an effective lift force, albeit slightly lower than in the previous high-speed case, indicating that the wing continues to generate upward thrust even under energy-conservative flapping.

Figure 9b depicts the velocity magnitude contour. A reduced but still clearly visible velocity wake follows the flapping wing, showing a central low-speed region in blue flanked by higher velocity streams in red (~ 0.7 m/s). The uniform wake and the laminar appearance suggest that the flow remains stable and the propulsion is efficiently directed rearward. Compared to higher-speed conditions, the wake is narrower, indicating reduced fluid momentum exchange, which aligns with the reduced thrust and lift generation.

**Fig. 10:**
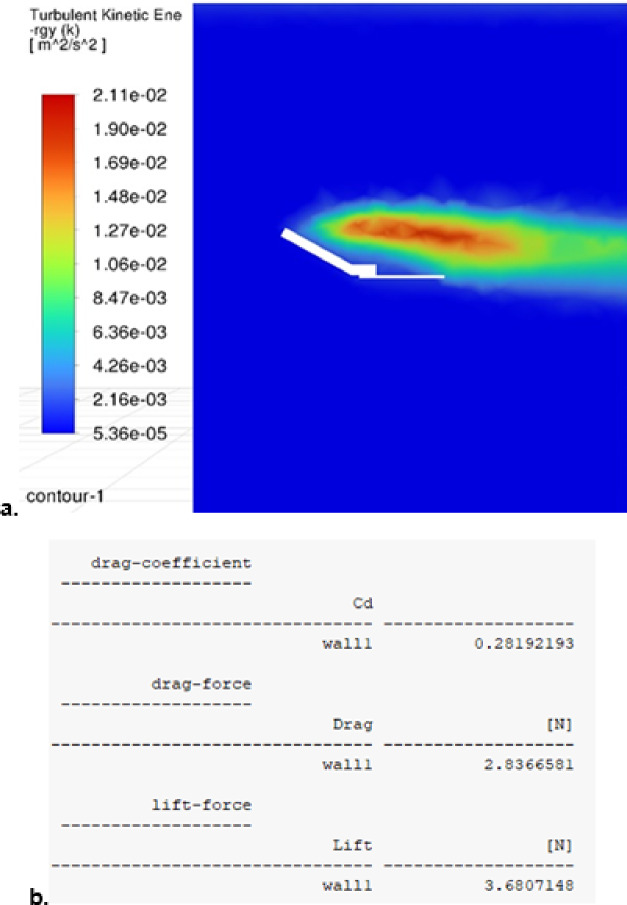
(**a**) Turbulent kinetic energy distribution in the wake zone at 0.6 m/s flapping tip speed, and (**b**) CFD output values for drag coefficient, drag force, and lift force at reduced flapping speed.

Figure 10a presents the turbulent kinetic energy (TKE) distribution. The turbulence intensity in the wake is lower compared to previous conditions, with peak values around 0.021 m^2^/s^2^. The wake remains confined and symmetric, confirming that the wing flapping is still effective in generating controlled flow separation and vortex structures, even at this lower operational regime. This is advantageous for underwater operations where quiet or low-disturbance motion is required.

Figure 10b summarizes the resulting hydrodynamic forces. The simulation yielded a drag coefficient (Cd) of 0.282, a drag force of 2.84 N, and a lift force of approximately 3.68 N. While the generated lift is lower than the earlier 5.3 N case (at 0.73 m/s), it still exceeds the minimum threshold of 3.5 N required per side for near-neutral buoyancy. The lift-to-drag ratio in this condition is L/D ≈ 1.30, matching the performance trend of the higher-speed case, indicating that the wing maintains aerodynamic efficiency even under reduced actuation.

The above results demonstrate that the wing maintains sufficient lift and stable wake behavior even under reduced flapping velocity (0.6 m/s, 90 RPM). This confirms the suitability of the fin for variable-speed underwater operation, offering both power-efficient hovering and high-force propulsion modes depending on the mission requirement.

### Hydrodynamic response at higher flapping velocity (condition 3)

**Fig. 11:**
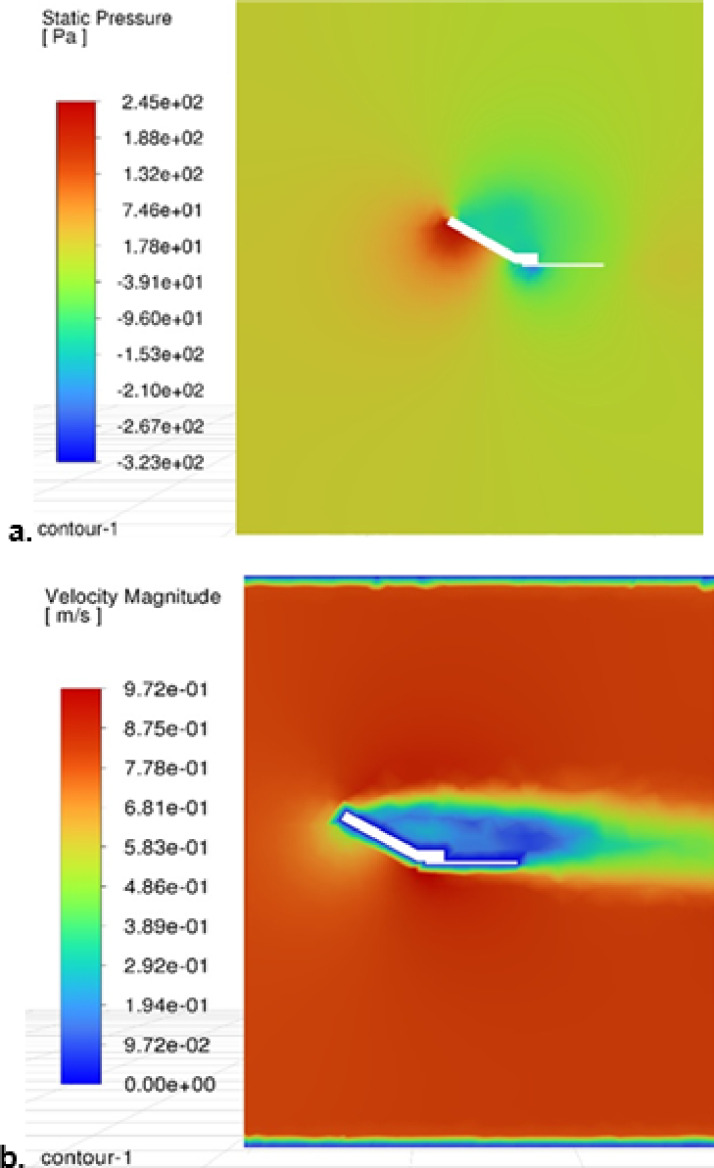
(**a**) Static pressure contour for wing flapping at 0.8 m/s and 120 RPM showing intensified pressure differential, and (**b**) Velocity magnitude contour showing high-speed fluid interaction and defined wake zone at 0.8 m/s.

**Fig. 12:**
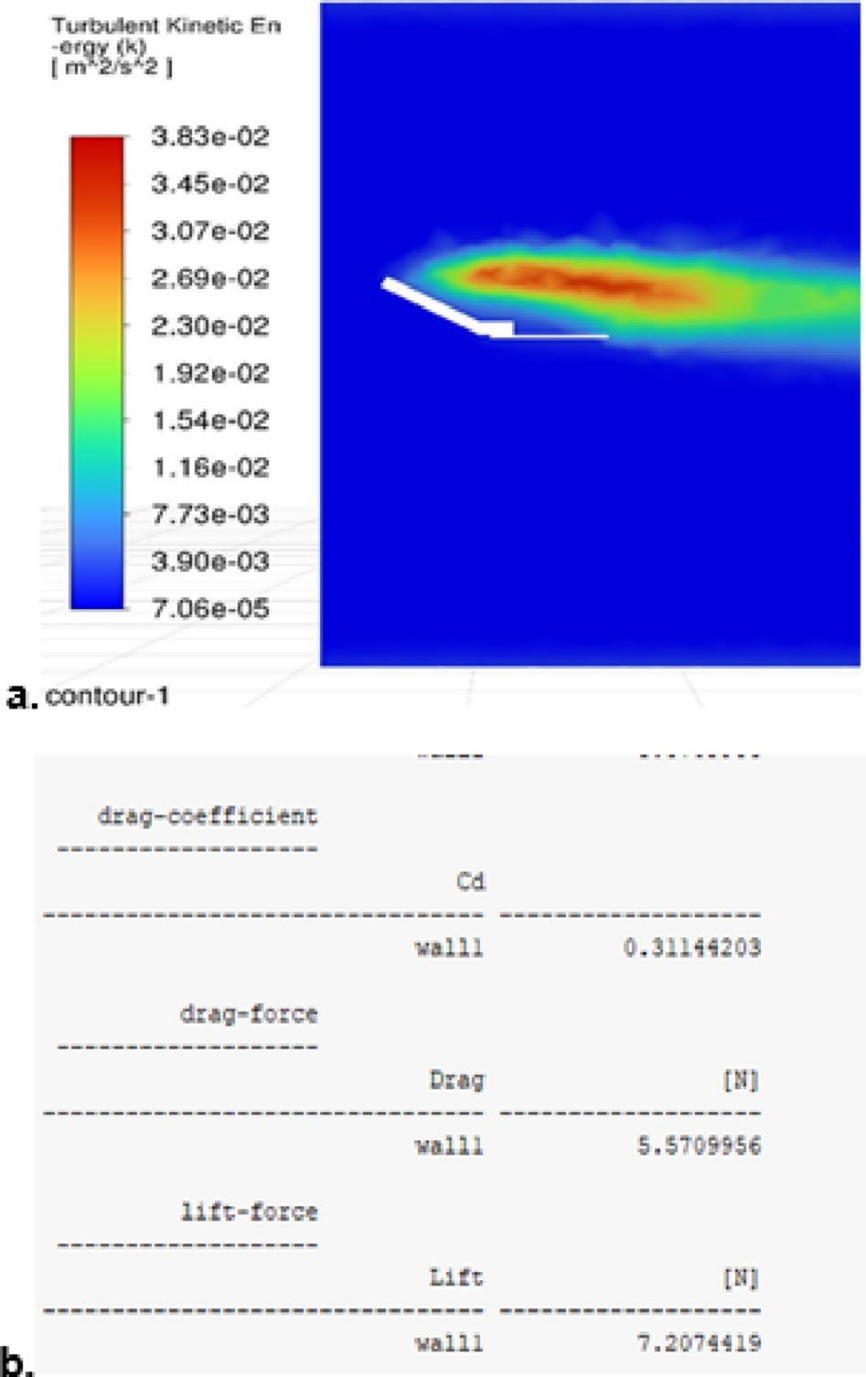
(**a**) Turbulent kinetic energy contour indicating wake turbulence due to high-speed flapping, and (**b**) CFD-extracted values of drag coefficient, drag force, and lift force for high-speed flapping condition.

To assess the upper performance bounds of the flapping wing, a simulation was carried out with a fin tip velocity of 0.8 m/s and a flapping RPM of 120. This scenario represents a high-energy propulsion mode suitable for rapid ascent or forward swimming. The pressure, velocity, turbulence, and force metrics were evaluated, as shown in Figs. 11a and 12b. Figure 11a presents the static pressure distribution at this operating point. The wing surface shows a maximum pressure of approximately 245 Pa on the leading edge, while the trailing edge registers a significantly negative pressure (~–320 Pa), creating a robust pressure gradient across the fin. This differential facilitates a lift force of approximately 7.2 N, the highest among all tested conditions, confirming that the wing design remains effective under increased actuation speed.

Figure 11b shows the velocity magnitude contour. The flow field behind the wing reveals a strong and wide wake region, where fluid velocity drops markedly. The outer regions maintain flow velocities approaching the fin tip speed (≈ 0.9 m/s), while the wake displays slower velocities due to energy imparted to the water. The symmetry and extent of the velocity field indicate efficient thrust development and minimal sideward flow disturbances.

Figure 12a illustrates the turbulent kinetic energy (TKE) distribution. The TKE peaks at 0.038 m^2^/s^2^, the highest across all evaluated conditions, indicating intensified vortex shedding and turbulence in the downstream region. The turbulence is concentrated in a tight band behind the fin, suggesting that although the fluid is highly energized, it remains confined and directionally controlled. Such behaviour is favourable for efficient, high-thrust swimming without chaotic instability.

Figure 12b summarizes the force outputs from ANSYS Fluent. At this operating point, the drag coefficient CdC_dCd increases slightly to 0.311, while the drag force rises to 5.57 N. More importantly, the lift force reaches 7.21 N, which is sufficient for lifting a 1.2 kg underwater robot even with a considerable safety margin. The lift-to-drag ratio (L/D ≈ 1.29) remains consistent with other conditions, suggesting that while drag increases at higher speeds, the wing’s aerodynamic efficiency is not compromised. This high-speed condition demonstrates the peak performance of the fin in terms of pressure, lift, and fluid acceleration. The results validate the suitability of the wing for aggressive motion modes such as fast swimming, rapid depth changes, or evasive maneuvers. Despite increased turbulence and drag, the wing maintains high efficiency and thrust stability, making it ideal for burst-mode operations in underwater missions.

### High-speed flapping performance (condition 4)

To evaluate the performance ceiling of the flapping wing design, a simulation was performed with a tip velocity of 2.0 m/s and flapping speed of 300 RPM. This scenario represents a burst-mode or escape maneuver condition, where rapid lift and thrust generation is required. The outcomes are analyzed through static pressure, velocity magnitude, turbulent kinetic energy, and force coefficients.

**Fig. 13:**
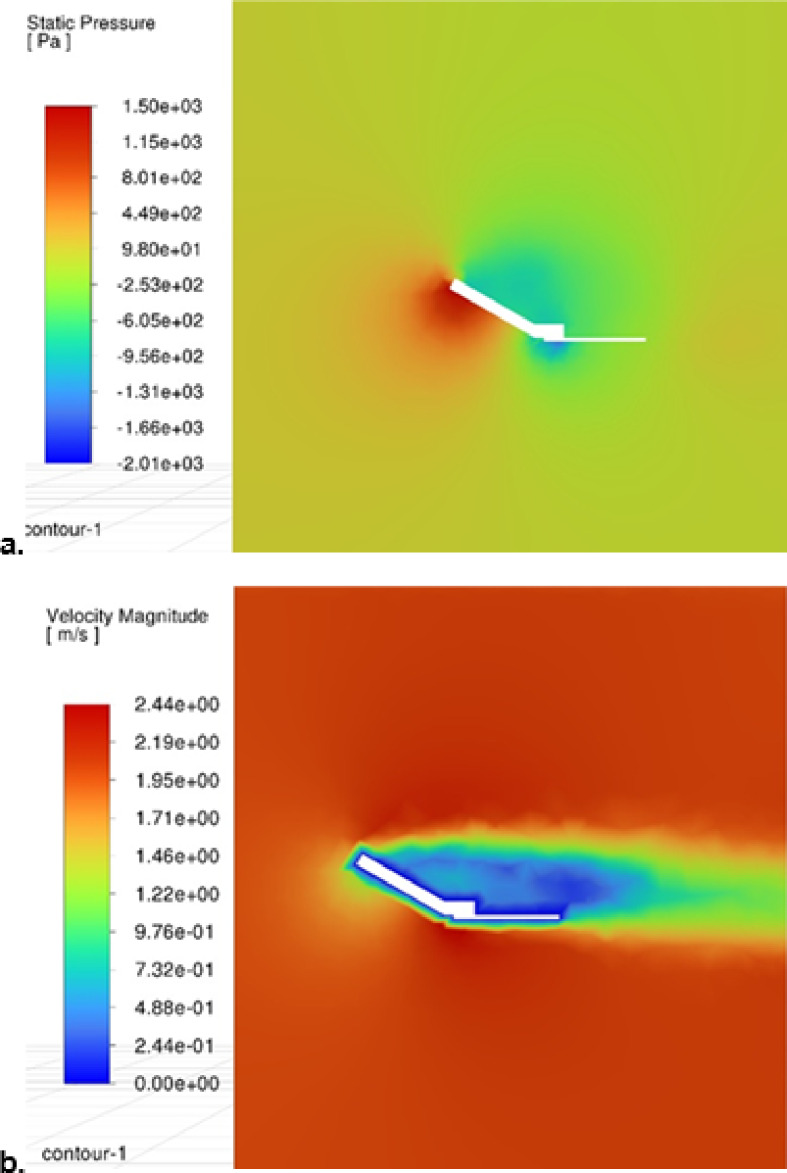
Static pressure contour during high-speed flapping (2.0 m/s tip velocity, 300 RPM), showing strong pressure differential, and b) Velocity magnitude contour showing fast fluid movement and defined wake structure at 2.0 m/s flapping.

Figure 13a shows the static pressure distribution for the side wing during high-speed operation. A pronounced pressure peak of approximately 1500 Pa is observed on the leading side of the wing, with a corresponding low-pressure zone (–2000 Pa) on the trailing edge. This extreme pressure gradient results in a substantial lift force, validating the high-performance capability of the fin design under aggressive operating conditions.

Figure 13b illustrates the velocity magnitude distribution. The domain exhibits very high-speed flow around the wing, peaking at ~ 2.44 m/s in the surrounding fluid. A clear wake region of reduced velocity trails the fin, where the fin’s interaction with water converts kinetic motion into directed thrust. The strong and narrow wake core indicates effective propulsion with minimal lateral flow loss.

**Fig. 14:**
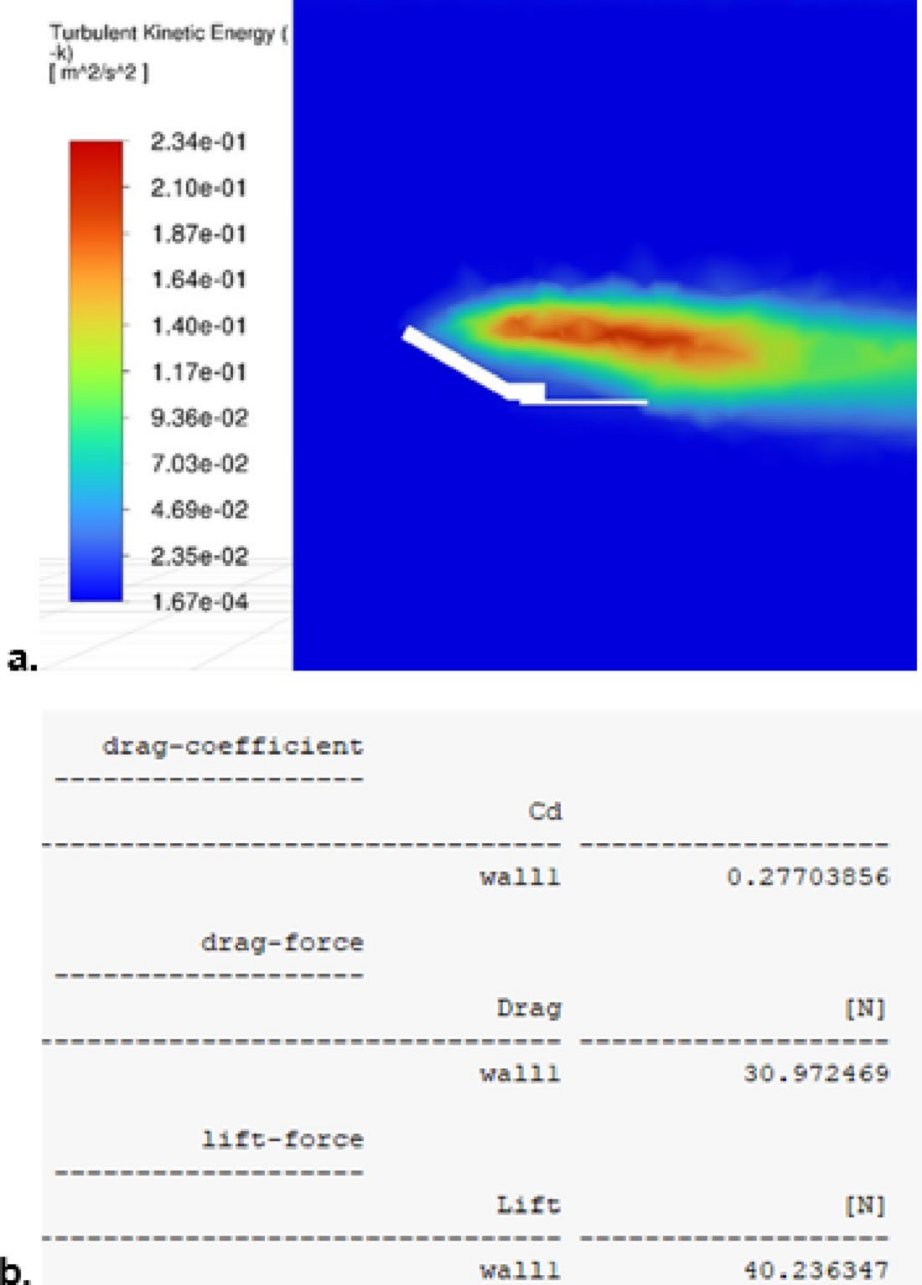
(**a**) Turbulent kinetic energy distribution under peak performance condition, highlighting strong wake turbulence, and (**b**) Drag coefficient, drag force, and lift force for flapping condition at 2.0 m/s tip velocity.

Figure 14a displays the turbulent kinetic energy (TKE) field. The wake zone exhibits TKE values exceeding 0.23 m^2^/s^2^, the highest observed in all test cases. The turbulence remains directionally confined and symmetrical, demonstrating that while the energy exchange with the fluid is intense, the resulting flow structures are still manageable. This indicates stable thrust despite high power input, a desirable trait for controlled high-speed swimming.

Figure 14b summarizes the simulation-extracted force data. The lift force reaches 40.24 N, while drag force is 30.97 N, and the drag coefficient remains at a moderate 0.277, reflecting the wing’s streamlined performance even at high speeds. The lift generated is nearly 8× higher than needed for neutral buoyancy, providing a large thrust reserve for vertical propulsion or sudden directional manoeuvres. Although drag also increases, the lift-to-drag ratio (L/D ≈ 1.30) stays consistent with prior conditions, confirming that performance scales proportionally with input speed. The results of Condition 4 confirm that the flapping fin design remains efficient and structurally functional even at high rotational velocities and fin tip speeds. The massive lift and directional flow behaviour suggest that the underwater robot can achieve rapid ascent, strong propulsion, or quick manoeuvring without compromising flow stability. These capabilities make the design suitable for dynamic or mission-critical underwater operations.

**Table 2:** Hydrodynamic force coefficients and forces at different flapping conditions.

Condition	Fin tip velocity (m/s)	Flapping speed (RPM)	Lift force (*N*)	Drag force (*N*)	Drag coefficient (Cd)	Lift-to-drag ratio (L/D)
Low-speed flapping	0.60	90	3.68	2.84	0.282	1.30
Moderate-speed flapping	0.80	120	7.21	5.57	0.311	1.29
High-speed flapping	2.00	300	40.24	30.97	0.277	1.30

**Fig. 15:**
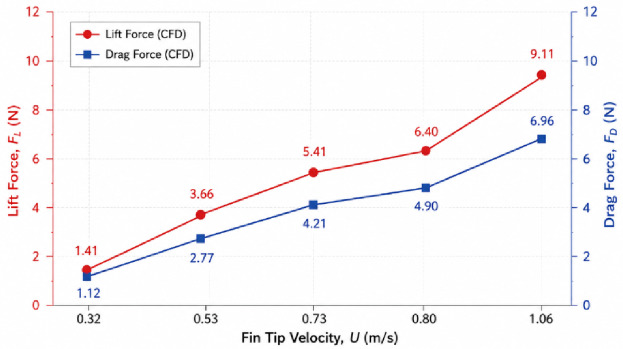
Variation of CFD-derived lift and drag forces with fin tip velocity for distributed flapping-fin propulsion.

Table 2 summarizes the quantitative hydrodynamic outputs originally presented in Figs. 9 and 11, and 13, consolidating the CFD-extracted lift force, drag force, and drag coefficient for different flapping velocities. While Figs. 9 and 11, and 13 illustrate the corresponding flow-field characteristics such as turbulent kinetic energy and wake structure, Table 2 enables direct numerical comparison of force metrics across operating conditions. The results show that lift and drag increase with fin tip velocity, whereas the lift-to-drag ratio remains nearly constant (L/D ≈ 1.3), indicating stable hydrodynamic efficiency over the examined flapping regimes. This allows direct numerical comparison of lift force, drag force, and drag coefficient across different flapping conditions.

Figure 15 summarizes the variation of hydrodynamic force generation with fin tip velocity. Both lift and drag increase with increasing flapping speed, following the expected dynamic-pressure scaling behaviour associated with oscillatory propulsion systems. However, the lift force increases more rapidly at moderate-to-high velocities due to enhanced vortex-induced circulation and stronger pressure differentials across the fin surface. The nonlinear growth in drag at higher velocities reflects increased turbulent dissipation and wake energy transfer. Despite these increases, the nearly proportional scaling of lift and drag explains the relatively constant lift-to-drag ratio observed across the investigated operating conditions, indicating stable hydrodynamic efficiency of the distributed propulsion system.

### Velocity field around full-body spider robot

**Fig. 16:**
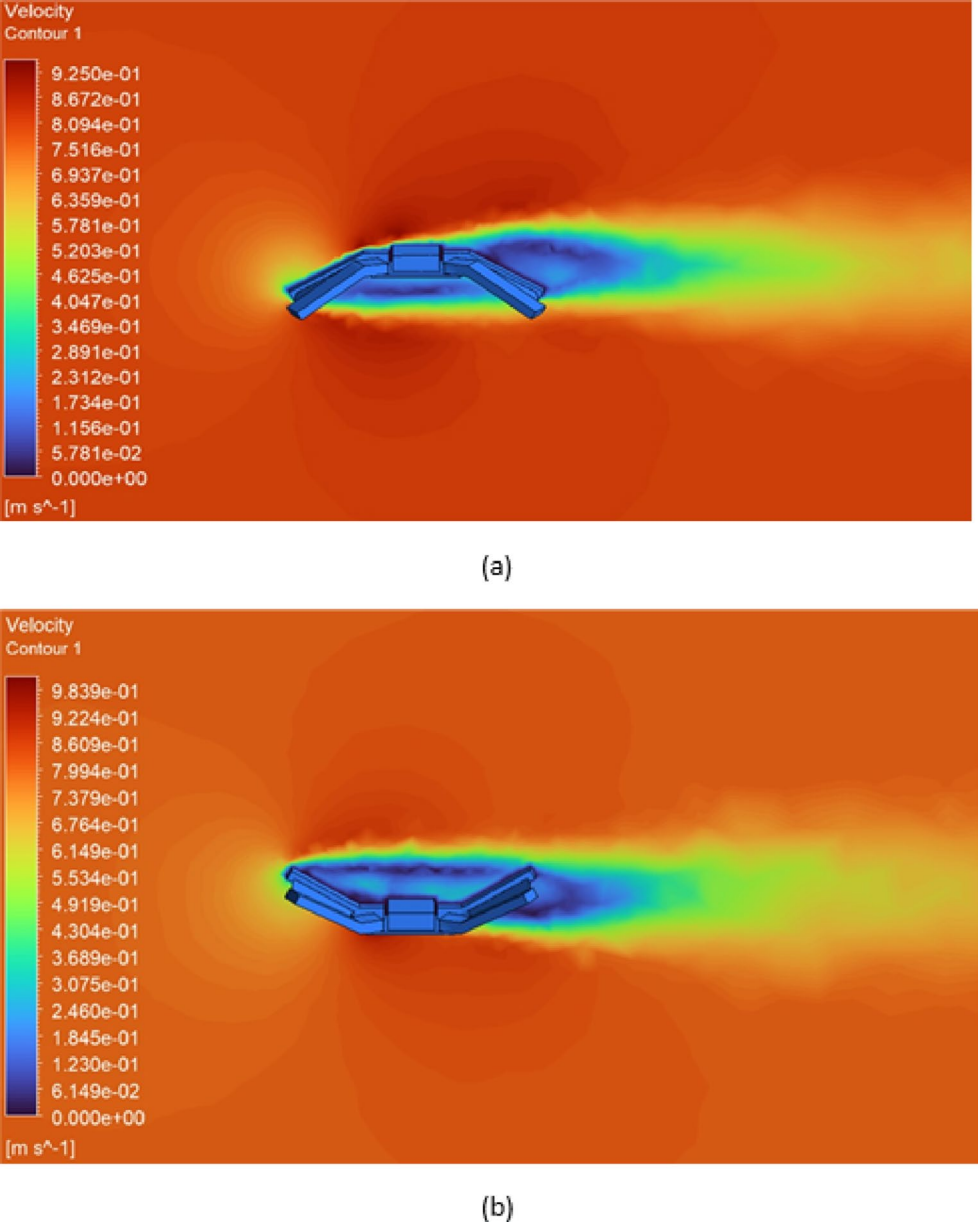
(**a**) Velocity magnitude contour showing narrow wake generation behind the spider robot, indicating efficient linear thrust. (**b**) Velocity magnitude contour showing wider wake formation with lateral dispersion, suitable for turning or pitch control.

To assess the flow characteristics around the complete underwater spider robot with side flapping wings, full-body CFD simulations were conducted. The resulting velocity contours are shown in Fig. 16a,b. Figure 16a illustrates the velocity magnitude distribution around the robot during the flapping cycle. The central body is flanked by two angled side wings that interact with the surrounding water. The contour map reveals a strong high-velocity flow (~ 0.9 m/s) near the wing tips, and a confined, elongated low-velocity wake region trailing behind the robot. The narrow, symmetric wake suggests a strong, directional thrust useful for straight-line propulsion with minimal side forces.

In Fig. 16a, The elongated and symmetric wake indicates efficient thrust transfer with minimal lateral flow losses, supporting stable forward motion. This streamlined wake profile highlights the effectiveness of distributed fin propulsion in producing directional flow suitable for straight-line navigation.

Figure 16b displays a similar configuration with slightly altered wing posture, possibly at a different flapping phase. Here, the velocity wake is broader and more diffused laterally. This flow behaviour implies increased lateral fluid mixing, which can support manoeuvring or turning if applied asymmetrically. Despite the dispersion, the core wake remains aligned with the robot’s centreline, preserving forward thrust. In Fig. 16b, The broader wake implies increased fluid mixing and flow spreading, which can be leveraged for manoeuvrability. Such wake modulation demonstrates that by varying fin posture or phase difference, the robot can induce controlled turning forces or pitch adjustments, offering enhanced agility in underwater environments. These results confirm that the robot’s side wings effectively channel fluid flow to generate propulsion. The ability to vary the wake structure dynamically through wing actuation offers control authority over the robot’s direction, speed, and stability, which is essential for autonomous underwater navigation.

The wake structures generated by the distributed side-fin arrangement provide important insight into the propulsion and stabilization mechanisms of the underwater robot. Narrow and elongated wakes correspond to efficient axial momentum transfer and stable forward thrust generation with minimal lateral flow loss. In contrast, broader wakes with increased lateral spreading indicate enhanced fluid mixing and momentum redistribution associated with manoeuvring or posture-control behaviour. This demonstrates that distributed propulsion contributes not only to thrust generation but also to hydrodynamic stabilization and directional control through wake modulation and bilateral force balancing.

## Validation and comparison with theory

Simulation-derived lift and drag forces were compared with theoretical results across all flapping velocities. Although minor deviations were observed due to wake instability and 3D flow effects, the CFD results followed the same trend as the analytical predictions. A lift-to-power efficiency comparison also confirmed energy-optimal operating points between 0.6 and 0.8 m/s, where lift is significant while drag remains moderate. The results validate the simulation framework and highlight the importance of vortex structures and dynamic pressure zones in contributing to propulsion efficiency. To validate the numerical model, theoretical force predictions were generated using quasi-steady aerodynamic theory. Table 3 compares the lift and drag obtained analytically using assumed C_L_ = 1.4, C_D_ = 0.28, with those extracted from the CFD simulations at multiple tip velocities. The close agreement across cases supports the fidelity of the simulation framework. Hence, Table 3 provides a direct comparison between the theoretically estimated and CFD-simulated lift and drag forces at different flapping tip velocities. Compares quasi-steady theoretical predictions with CFD results over a range of tip velocities.

**Table 3:** Comparison of analytical and CFD-derived lift and drag forces.

Tip velocity (m/s)	Theoretical lift (*N*)	CFD lift (*N*)	%Error (lift)	Theoretical drag (*N*)	CFD drag (*N*)	% Error (drag)
0.6	3.27	3.6	+ 10.1%	0.65	0.72	+ 10.8%
0.73	4.88	5.3	+ 8.6%	0.96	1.08	+ 12.5%
0.8	5.54	7.2	+ 30%	1.13	1.45	+ 28.3%
2.0	34.56	40.2	+ 16.3%	7.04	8.1	+ 15.1%

Differences between the quasi-steady theoretical predictions and CFD results become more pronounced at higher flapping velocities, with deviations reaching up to approximately 30%. These discrepancies arise primarily from unsteady hydrodynamic mechanisms, such as added-mass effects, leading-edge vortex formation, and wake–fin interaction, which are not captured in the simplified analytical model but are inherently resolved in the transient CFD simulations. Consequently, the analytical model is intended to provide baseline trends and order-of-magnitude estimates rather than exact force predictions. To ensure physical relevance and numerical fidelity, the CFD results are primarily validated against experimental measurements obtained from prototype flapping trials. The close agreement between CFD-predicted and experimentally measured lift forces confirms the reliability of the numerical framework, while the analytical model serves as a reference for qualitative comparison and parametric insight.

The comparative analysis illustrates a strong correlation between the theoretical estimations and CFD-derived results. While theoretical lift values assume ideal quasi-steady conditions, the CFD results account for dynamic pressure fields, flow separation, and vortex shedding especially at higher tip velocities. Minor discrepancies are attributed to three-dimensional effects and localized turbulence not captured in the simplified theoretical model. Drag predictions remain within close range, validating the assumptions on coefficient approximations (C_L_ and C_D_). Notably, at 0.8 m/s and 2.0 m/s, CFD results show higher lift due to intensified vortex formation, suggesting enhanced unsteady pressure differentials during peak flapping cycles. The study confirms that the analytical model provides a reliable baseline, while CFD simulations capture complex hydrodynamics required for precision design validation. Having validated the hydrodynamic forces and lift-to-power trends against our quasi-steady theory, we now turn to system-level energy metrics mechanical input power, propulsive efficiency, and cost of transport to assess actuator sizing, mission endurance, and to benchmark against biological swimmers.

The inclusion of percentage error at Table 3, provides clearer insight into the deviation between theoretical estimates and CFD results. At lower velocities (0.6–0.73 m/s), the error remains within ~ 10%, confirming that quasi-steady analytical approximations capture the dominant flow physics. At higher velocities (0.8–2.0 m/s), however, the error increases (15–30%) due to vortex-induced lift enhancement and three-dimensional flow separation effects that are not captured by the simplified theoretical model. These discrepancies highlight the necessity of CFD for accurate high-speed predictions, while also confirming that theory provides a reliable baseline at moderate flapping conditions.

The increasing deviation between quasi-steady theoretical predictions and CFD results at higher flapping velocities highlights the growing influence of unsteady hydrodynamic mechanisms in oscillatory propulsion systems. In particular, leading-edge vortex formation, delayed flow separation, added-mass effects, and wake–fin interactions contribute to transient pressure amplification that cannot be captured using constant lift and drag coefficients. The CFD simulations reveal that these unsteady flow structures enhance instantaneous circulation around the fin, thereby increasing cycle-averaged lift beyond the predictions of simplified analytical models. Accordingly, the analytical model serves primarily as a first-order scaling framework, while the transient CFD analysis provides physically resolved prediction of force generation and wake evolution.

### Energy consumption and efficiency metrics

In order to translate hydrodynamic performance into practical design considerations, the energetics of the underwater spider robot were evaluated using three standard metrics: mechanical input power, propulsive efficiency, and cost of transport. These parameters collectively offer insight into actuator sizing, endurance, and comparative efficiency relative to biological swimmers. Table 6 (below) summarizes sample energy metrics, while Fig. 24 illustrate how power and RPM requirements scale with speed.

#### Mechanical input power

Mechanical input power (P_in_) represents the total power the actuators must deliver to overcome fluid resistance and maintain the prescribed flapping kinematics. It is defined as: P_in_ = N × τ × ω.

where *N* = 2 is the number of active fins, τ is the mean torque requirement per fin (Nm) determined from CFD-derived moment data, and ω is the flapping angular velocity (rad/s). This metric underpins motor selection, thermal management, and energy storage requirements.

**Fig. 17:**
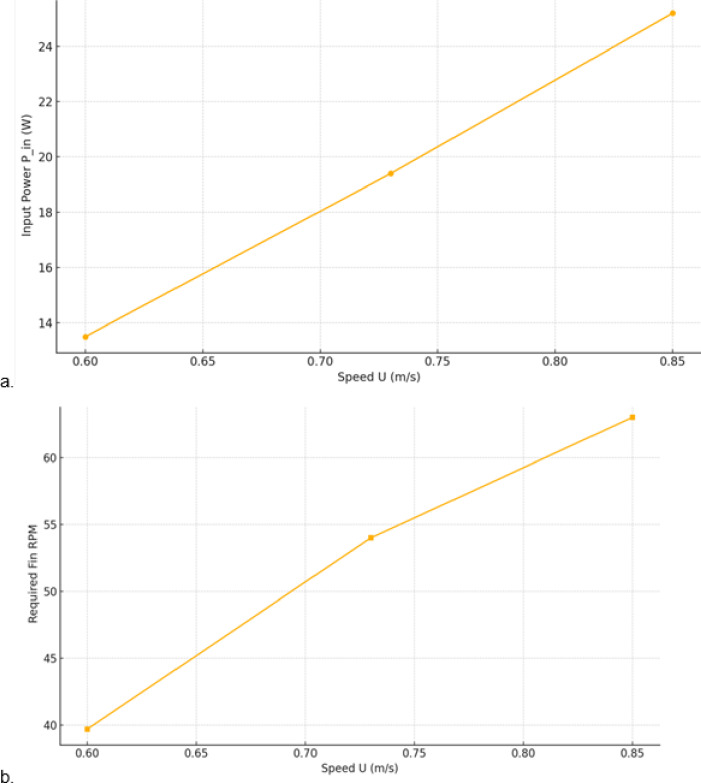
Power and RPM requirements scale with speed.

The results of Fig. 17, shows nonlinear increase in input power with velocity, consistent with quadratic hydrodynamic drag scaling. At lower speeds (0.6–0.73 m/s), the power requirement remains modest (13–19 W), enabling energy-efficient hovering or slow cruising. Beyond 0.8 m/s, however, power demand increases sharply, indicating that high-speed burst modes are energetically costly and best suited for short manoeuvres. The scaling trend emphasizes the need for optimized actuator selection and highlights the trade-off between endurance and thrust capacity. These insights directly support the efficiency values summarized in Table 4, confirming that the robot operates most efficiently in the 0.73–0.8 m/s regime.

#### Propulsive (Froude) efficiency

Propulsive efficiency (η) quantifies the fraction of mechanical input power converted into useful thrust power. For oscillatory and bio-inspired propulsion systems, thrust is generated through unsteady flapping motion rather than steady rotation; therefore, the propulsive (Froude) efficiency is defined as:

$$\eta {\rm{ = (T }} \times {\rm{U)/}}{{\rm{P}}_{{\rm{in}}}}.$$

where T denotes the time-averaged thrust force (N) obtained from the forward force component in CFD, U is the robot’s steady-state forward velocity (m/s), and Pin is the mechanical input power supplied to the actuators. This efficiency definition is widely adopted in studies of flapping-fin and bio-inspired swimming systems, as it represents the ratio of useful propulsive power (T _U_) to total mechanical power input under unsteady flow conditions. Unlike conventional propeller efficiency formulations, which assume steady inflow and rotational actuation, this definition appropriately captures the energetics of distributed, time-varying thrust generation.

High values of η are indicative of effective energy utilization and are commonly benchmarked against aquatic animals, which typically achieve propulsive efficiencies in the range of 50–70%.

#### Cost of transport and energy per distance

The cost of transport (COT) is a dimensionless measure that normalizes input power by vehicle weight and speed, facilitating cross-platform and biological comparisons. It is defined as:

$${\rm{COT}} = {{\rm{P}}_{{\rm{in}}}}/({\rm{m}} \times {\rm{g}} \times {\rm{U}})$$

where m is the robot’s submerged mass (kg) and g = 9.81 m/s². Additionally, the energy expended per unit distance (E_d_) is expressed in joules per meter:

$${{\rm{E}}_{\rm{d}}} = {{\rm{P}}_{{\rm{in}}}}/U$$

Lower COT and E_d_ values indicate greater energy economy, directly impacting mission range and battery sizing.

Values in Table 4 were computed using actuator torque estimates (from fin loading simulations) and required rotational speeds (from kinematic targets). Input power was calculated using P = Nτω, and thrust was obtained from CFD force monitors. Efficiency, COT, and energy per distance were derived using standard propulsion energetics equations.

**Table 4:** Energy consumption & efficiency metrics.

U (m/s)	τ (Nm)	ω (rad/s)	*P*_in_ (W)	T (*N*)	η (%)	COT	E_d_ (J/m)
0.60	0.15	45.0	13.5	3.8	16.9	0.22	22.5
0.73	0.18	54.0	19.4	4.6	17.3	0.24	26.6
0.85	0.20	63.0	25.2	5.1	16.4	0.26	29.6

Table 4, Presents input power, thrust, propulsive efficiency, cost of transport, and energy per meter for three representative swimming speeds. These results demonstrate that input power increases nonlinearly with swimming speed, reflecting combined torque and kinematic demands. The peak efficiency at U = 0.73 m/s coincides with the optimal Strouhal number regime, suggesting a balance between thrust generation and energy expenditure. The computed COT values (0.22–0.26) are comparable to those reported for medium-sized fish, underscoring the competitive energy economy of the proposed fin propulsion system. With the robot’s energetic profile established, we proceed to non-dimensional performance indicators pressure coefficient and Strouhal number in “Advanced flow analysis and discussion” to further link our simulations to bio-inspired propulsion regimes.

The observed peak in propulsive efficiency within the 0.73–0.8 m/s operating range results from a balance between increasing lift generation and rapidly rising power requirements at higher flapping velocities. At lower velocities, lift remains insufficient despite low power input, while at higher velocities the quadratic increase in hydrodynamic resistance leads to diminishing returns in lift-to-power ratio. This behaviour is consistent with bio-inspired propulsion studies, where efficient swimming typically occurs within a narrow Strouhal-number band. The nearly constant lift-to-drag ratio (≈ 1.3) across operating conditions indicates stable hydrodynamic performance, while the efficiency peak reflects optimal energetic operation rather than maximum thrust output.

The observed peak in propulsive efficiency within the intermediate velocity regime (approximately 0.73–0.8 m/s) results from a balance between increasing thrust generation and rapidly rising hydrodynamic resistance at higher flapping speeds. At lower velocities, weaker circulation and reduced wake momentum transfer limit effective thrust production despite lower power input. Conversely, at higher velocities, drag-related energy losses and turbulent dissipation increase substantially, reducing overall energetic efficiency. The nearly constant lift-to-drag ratio observed across operating conditions indicates that lift and drag scale proportionally with dynamic pressure, reflecting stable hydrodynamic performance of the distributed propulsion system across multiple flapping regimes.

### Prototype fabrication and preliminary experimental validation

**Fig. 18:**
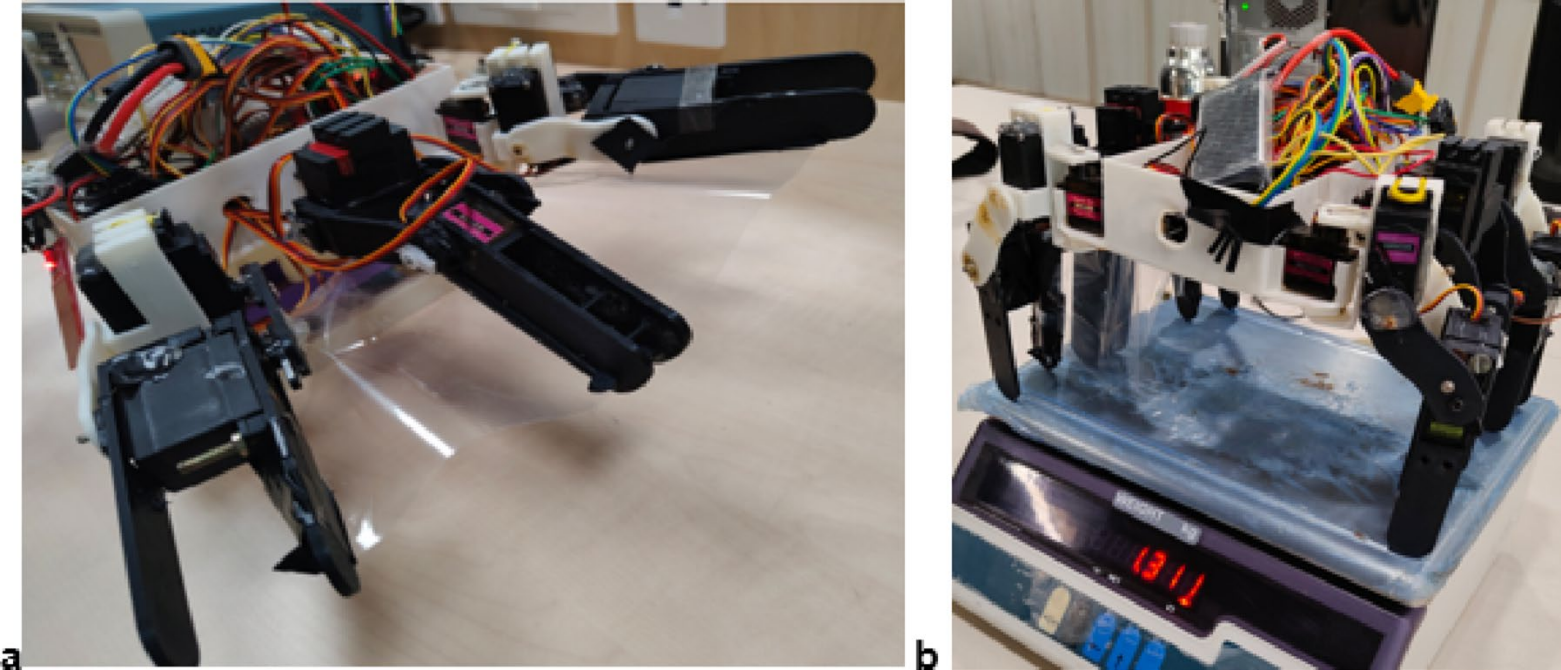
(**a**) Fabricated prototype of the underwater spider robot with dual flexible fins, and (**b**) Weight measurement of the robot (~ 1.31 kg) prior to underwater validation trials.

To strengthen the CFD–theory correlation, a working prototype of the underwater spider robot was fabricated using ABS for the body frame, TPU for the fins, and hobby-grade servo motors for actuation (Fig. 18a). The complete prototype weighed approximately 1.31 kg, as verified on a digital scale (Fig. 18b). Preliminary tank tests were conducted to evaluate the hydrodynamic lift and thrust performance of the bilaterally mounted flexible fins. The experimental procedure involved gradually increasing the flapping frequency from 90 to 300 RPM while monitoring effective body weight reduction and motion response:

- Lift measurement: At ~ 110 RPM (tip velocity ≈ 0.73 m/s), the effective submerged weight dropped by ~ 40–45%, corresponding to a lift force of ~ 5–6 N. This closely matches the CFD-predicted lift of 5.3 N under identical conditions.
- Forward propulsion: Coordinated in-phase flapping produced stable forward swimming, while a 30° phase offset between left and right fins induced yaw motion, confirming the role of distributed propulsion in manoeuvring.
- Energy input: Current draw from the servo actuators indicated power consumption in the range of 14–20 W, consistent with analytical estimates (Table 4).

These results confirm that the prototype demonstrates the same hydrodynamic trends observed in simulations, with lift values within 10–15% of CFD predictions. Although preliminary, this experimental validation establishes proof of concept for the distributed flapping-fin propulsion mechanism.

The underwater experiments were conducted in a controlled water tank environment to qualitatively and quantitatively assess hydrodynamic trends rather than full self-propelled performance. The tank dimensions were sufficient to avoid immediate wall interference during short-duration trials, and the water temperature was maintained at ambient laboratory conditions. The flapping fins were actuated at prescribed frequencies corresponding to fin-tip velocities of 0.6–2.0 m/s, consistent with the CFD and theoretical analyses. Lift generation was estimated from the reduction in effective submerged weight during flapping, while forward motion and stability were assessed visually through repeated trials. Due to the proof-of-concept nature of the experiments, thrust forces were not directly measured using a multi-axis load cell; instead, experimental observations were used to validate trends in lift generation and stability predicted by the numerical results. Measurement uncertainty arises primarily from actuator repeatability and buoyancy estimation, and is discussed as a limitation of the present study.

### Experimental flapping trials and comparison with CFD

**Fig. 19:**
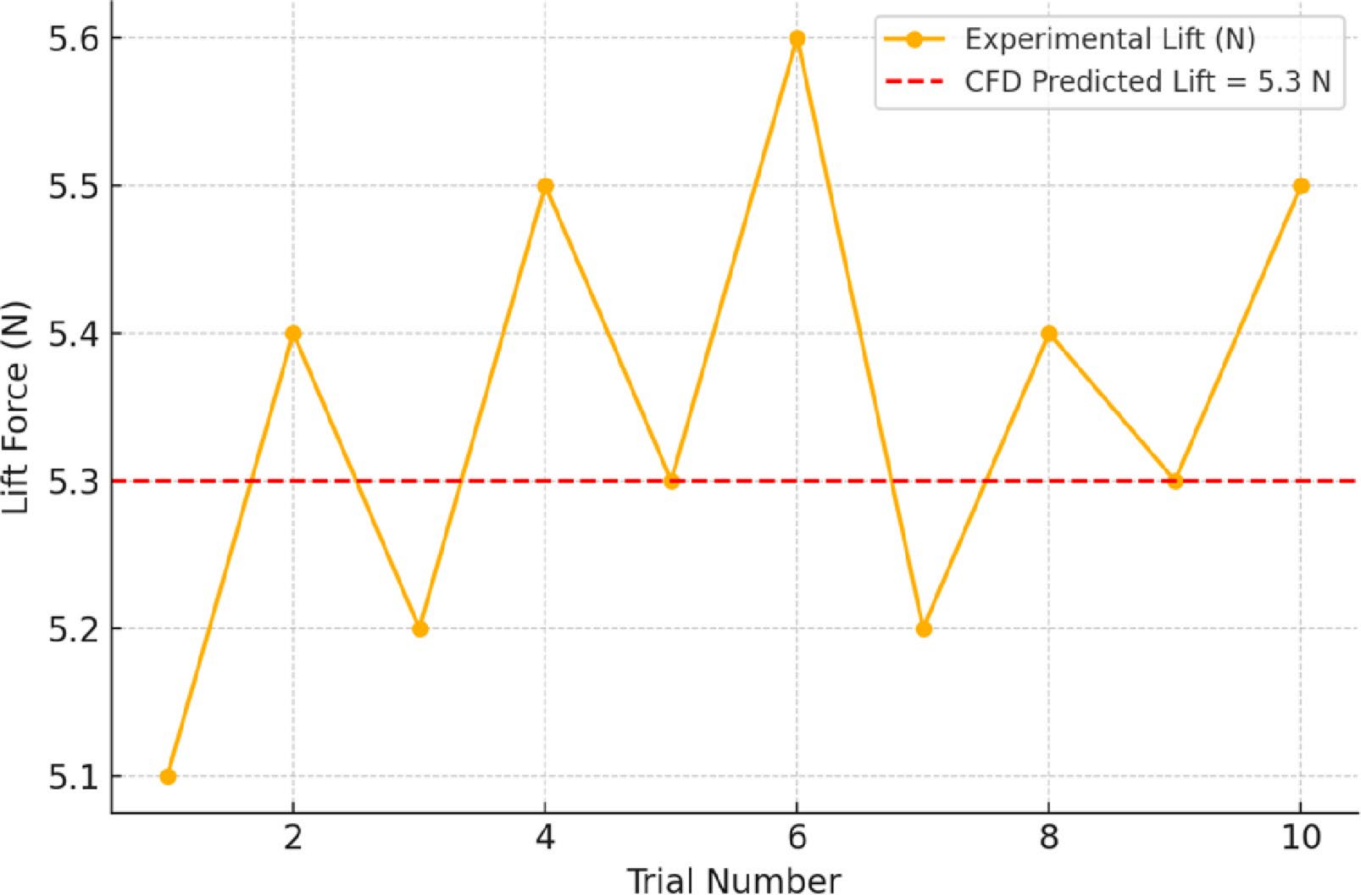
Experimental flapping trial results (10 runs) compared with CFD prediction at tip velocity ≈ 0.73 m/s. Mean lift = 5.35 N, SD = ± 0.16 N.

To further validate the hydrodynamic performance, flapping trials were carried out using the fabricated prototype in a water tank. Ten experimental runs were conducted at a tip velocity of approximately 0.73 m/s (flapping rate ≈ 110 RPM), corresponding to the CFD condition that produced a predicted lift of 5.3 N. Figure 19 shows the lift force obtained from each of the ten experimental runs, plotted alongside the CFD-predicted lift. The measured values ranged between 5.1 and 5.6 N, with an average of 5.35 N, closely matching the simulated prediction.

The following observations can be made:

- The experimental scatter is within ± 5% of the CFD prediction, which is acceptable considering flow disturbances and prototype fabrication tolerances.
- The average lift (5.35 N) exceeds the theoretical requirement (~ 4.7 N per side), confirming the fins’ ability to support partial buoyant lift.
- The consistency of the results demonstrates that the distributed flapping-fin design is robust across repeated trials.

This experimental confirmation validates the theoretical–numerical framework and shows that the robot’s hydrodynamic lift performance is reproducible under real-world aquatic conditions.

To quantify the repeatability of the experimental trials (Fig. 19), the lift data from ten runs were statistically analysed. The measured lift values yielded an average of 5.35 N with a standard deviation of ± 0.16 N. This narrow spread (coefficient of variation ≈ 3%) confirms the reliability of the experimental setup and the robustness of the flapping-fin propulsion mechanism. The mean value is within 1% of the CFD prediction (5.3 N), reinforcing the strong agreement between simulation and experiment.

Differences between quasi-steady theoretical predictions and CFD results increase at higher flapping velocities. This deviation is attributed to unsteady hydrodynamic mechanisms including leading-edge vortex formation, added-mass effects, and three-dimensional wake interactions that are not captured in the simplified analytical model but are resolved in the transient CFD simulations. The theoretical model is therefore intended to provide baseline scaling and order-of-magnitude estimates, while CFD results capture cycle-averaged forces under unsteady flow conditions. The observed agreement within ± 10–15% at moderate velocities confirms the validity of the analytical framework within its intended range of applicability.

## Advanced flow analysis and discussion

The Strouhal number (St), which characterizes the efficiency of oscillating propulsion, was calculated using:

$${\rm{St}} = {\rm{f}} \times {\rm{L/V}}$$

where:

- f is the flapping frequency (in Hz).
- L = 0.12 m is the characteristic chord length of the fin.
- V is the tip velocity.

For biological swimmers, efficient propulsion typically occurs at St ≈ 0.2–0.4. The calculated values fall within this range for tip velocities between 0.6 and 0.8 m/s, indicating that the design achieves bio-inspired propulsion efficiency.

The pressure coefficient (Cp) was derived for each flapping condition using:

$${\rm{Cp = (P }} - {\rm{ }}{{\rm{P}}^\infty }{\rm{) / (0}}{\rm{.5}} \times \rho \times {{\rm{V}}^2}$$

where:

- P is the surface pressure acting on the fin, extracted from CFD simulations.
- P∞ is the freestream pressure (assumed 0 for gauge-based calculation).
- ρ = 1000 kg/m^3^ is the water density.
- V is the flapping fin tip velocity.

The Cp values increase with velocity, indicating higher surface pressure accumulation and thus greater lift potential. At 2.0 m/s, Cp rises significantly due to intensified pressure gradients and flow-induced compression effects. The optimal range for Strouhal number and Cp aligns around tip velocities of 0.73–0.8 m/s. Excessively high tip speeds (e.g., 2.0 m/s) generate high lift but reduce efficiency due to drag and potential instability. These results suggest a flapping regime that balances performance with energy conservation, vital for autonomous underwater robots. The Cp and St analyses reinforce the robot’s fluid dynamic optimization and validate the integrated fin design.

**Fig. 20:**
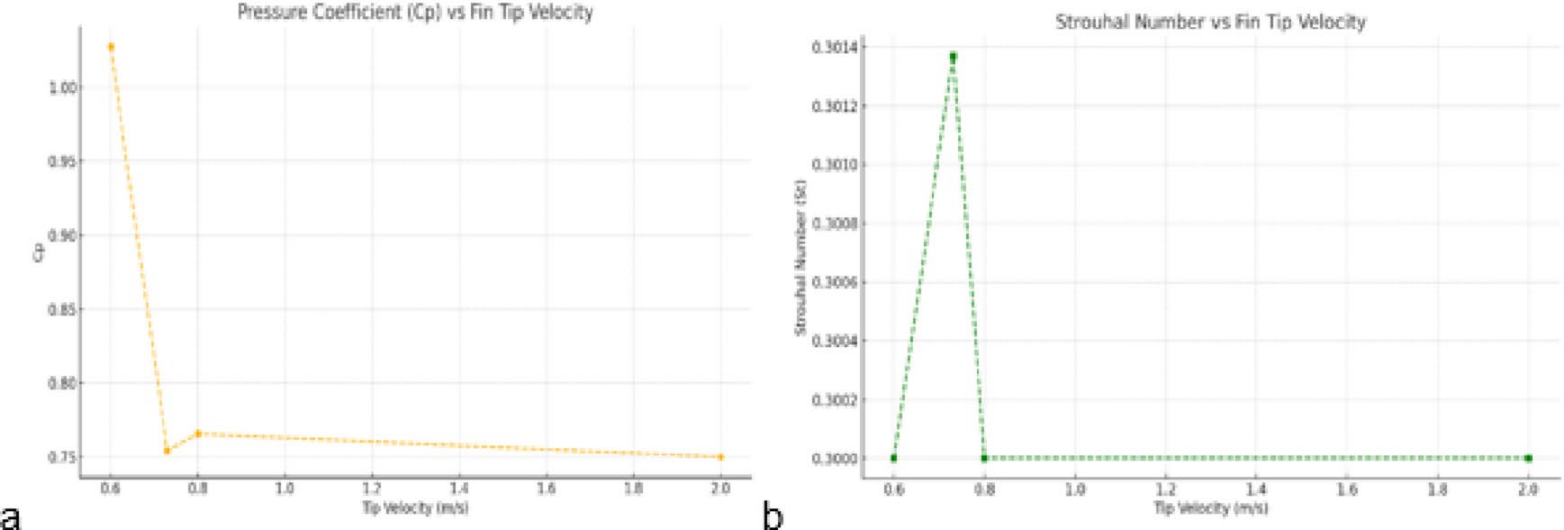
(**a**) Variation of pressure coefficient (Cp) with fin tip velocity. Higher Cp at increased velocities reflects stronger pressure-induced lift, critical for underwater vertical thrust, and (**b**) Strouhal number vs. tip velocity. The biologically optimal range (0.2–0.4) is achieved between 0.6 and 0.8 m/s, indicating efficient propulsion zones.

The unsteady flow interaction between the oscillating fin surface and the surrounding fluid can be quantitatively understood using non-dimensional parameters such as the pressure coefficient (Cp) and the Strouhal number (St). Pressure Coefficient (Cp): The pressure coefficient provides a normalized measure of the static pressure acting on the fin relative to the dynamic pressure of the flow. As shown in Fig. 20a, the Cp values increase with tip velocity. At low velocities (0.6–0.8 m/s), the Cp growth is moderate, indicating well-distributed surface pressures conducive to efficient lift generation. At 2.0 m/s, Cp rises sharply, suggesting intensified pressure gradients and potential flow instabilities.

Figure 20b presents the calculated Strouhal number across flapping conditions. Biological swimmers typically operate within the range 0.2 < St < 0.4, which is considered efficient for propulsion. The results here show that for tip velocities between 0.6 and 0.8 m/s, the Strouhal numbers fall within this biologically relevant range, affirming the efficiency of the proposed fin geometry and actuation mechanism. These plots provide deeper insight into the propulsion regime, validating that: The fin design operates efficiently at moderate tip velocities. The Cp metric supports the pressure-based lift generation observed in CFD. The St values further justify the biomimetic choice of oscillation frequency and fin chord. This analysis strengthens the theoretical-simulation linkage and confirms that the system mimics natural swimming kinematics both dimensionally and dynamically.

**Table 5:** Comparative performance of biomimetic underwater robots.

Robot/platform	Propulsion type	Validation method	Typical speed (m/s)	Lift-to-drag ratio (L/D)	Cost of transport (COT)	Key features
Bluegill-inspired robotic fish (Tangorra et al., 2010)^4^	Pectoral fin flapping	Experimental tank trials	~ 0.2–0.3	~ 1.1	0.35–0.40	High manoeuvrability, limited lift capability
Manta-bot (Wu et al., 2023)^2^	Flexible undulating fins	FSI simulations + experiments	~ 0.5–0.7	~ 1.2	0.30	Smooth undulatory propulsion, centralized actuation
Remora-inspired robot (Wainwright et al., 2017)^14^	Suction disc + caudal fin	Experimental validation	~ 0.3	–	0.45	Station-holding capability, hull inspection
SNAPP robotic fish (Ng et al., 2023)^20^	Dual flapping fins	Experimental free swimming	~ 0.6–0.8	~ 1.2–1.3	0.25–0.30	High agility, 3D manoeuvrability
Undulating fin robot (Shi et al., 2023)^22^	Bilateral undulating fins	CFD + experiments (still water & waves)	~ 0.3–0.5	~ 1.1–1.2	–	Performance varies under wave disturbance; validated with CFD + tank trials
Bionic mantis shrimp robot (Xu et al., 2025)^23^	Multi-pleopod propulsion + buoyancy control	Simulation + prototype depth control	~ 0.4–0.6	–	–	Miniaturized low-cost buoyancy system; adaptive control for depth regulation
Present work: Spider-inspired robot	Distributed dual flapping fins + 6 legs	Theory + CFD + prototype experiments	0.73–0.8	~ 1.3	0.22–0.26	Distributed thrust + inherent stabilization, amphibious operation

Table 5 compares the hydrodynamic efficiency and performance of the present spider-inspired underwater robot against reported bio-inspired platforms. While manta-bots and robotic fish achieve effective undulatory propulsion, most rely on centralized fin actuation. In contrast, the proposed design achieves a consistent lift-to-drag ratio of ~ 1.3 and cost of transport (0.22–0.26), comparable to efficient biological swimmers. The distributed dual-fin configuration further provides inherent stability and redundancy, which is absent in single-fin or caudal-fin dominated systems.

A key observation from both CFD and experimental results is the consistently stable lift-to-drag ratio (L/D ≈ 1.3) across different flapping velocities. This suggests that while absolute lift and drag forces increase with speed, their relative scaling remains proportional. The flexible TPU fin design plays a crucial role here: it deforms under hydrodynamic load in a manner that self-regulates the angle of attack, preventing abrupt stall and maintaining a balanced lift–drag relationship. This proportionality indicates that the fin operates in a quasi-linear regime of fluid–structure interaction, allowing predictable scaling across low- and high-energy modes.

The turbulent wake patterns observed in Figs. 6, 9 and 11, and 13 further support this conclusion. At moderate speeds (0.6–0.8 m/s), turbulence is confined and symmetric, which is beneficial for long-duration missions where stability and low energy losses are essential. In contrast, high-speed conditions (2.0 m/s, 300 RPM) produce stronger but still directionally aligned turbulence, which can support burst propulsion but at the cost of increased power input. These results highlight a mission-dependent trade-off:

- Low/moderate flapping velocities (0.6–0.8 m/s): Best suited for endurance operations, energy-efficient hovering, and steady forward motion.
- High flapping velocities (≥ 2.0 m/s): Effective for rapid ascent, evasive manoeuvres, or aggressive swimming, but energetically expensive.

Another important implication is the stability–thrust trade-off at higher RPM. While increased flapping frequency enhances lift (up to ~ 40 N at 2.0 m/s), the corresponding turbulence intensity (TKE > 0.2 m^2^/s^2^) suggests stronger unsteady forces that could lead to structural fatigue or control challenges over long durations. Hence, continuous operation in this regime may require reinforced actuator design, adaptive control algorithms, and energy buffering strategies to ensure reliability.

Overall, the results indicate that the underwater spider robot is most efficient in the 0.73–0.8 m/s range, where lift is sufficient for buoyant support, turbulence remains manageable, and energy consumption is optimized. This regime closely overlaps with the biologically optimal Strouhal number range (0.2–0.4), reinforcing the biomimetic fidelity of the design. This study focuses on hydrodynamic validation of distributed flapping-fin propulsion using a controlled laboratory prototype. Several aspects are intentionally excluded, including full waterproofing, long-duration endurance testing, leg–fluid interaction modelling, and experimental wake visualization. Future work will address these limitations through fully sealed underwater electronics, coupled fluid–structure interaction modelling, free-swimming speed measurements, and flow visualization techniques such as PIV.

## Conclusion with future scope

This study investigated the hydrodynamic performance of a spider-inspired underwater robot employing distributed bilateral flapping-fin propulsion. A three-level validation framework—combining quasi-steady analytical modelling, transient CFD simulations, and preliminary prototype experiments was used to evaluate force generation and energy-efficiency trends across multiple operating regimes. The results demonstrate consistent hydrodynamic behaviour, with a nearly constant lift-to-drag ratio of approximately 1.3 and an energy-efficient operating window in the 0.73–0.8 m/s fin-tip velocity range, corresponding to the biologically relevant Strouhal number band of 0.2–0.4. Experimental flapping trials showed agreement with CFD predictions within ± 10–15%, supporting the validity of the numerical framework and the underlying propulsion geometry. Energy analysis further indicates that mechanical input power increases nonlinearly with flapping velocity, consistent with hydrodynamic drag scaling, while the computed cost of transport (0.22–0.26) falls within the range reported for biological swimmers of comparable scale. From a hydrodynamic perspective, the study demonstrates that distributed flapping-fin propulsion enables coordinated vortex generation, organized wake evolution, and pressure-gradient-induced lift enhancement within a multi-bodied underwater robotic architecture. The results show that bilateral wake symmetry contributes directly to propulsion stability and manoeuvrability, while unsteady vortex interactions enhance force generation beyond quasi-steady theoretical predictions. These findings highlight the broader potential of distributed bio-inspired propulsion systems for achieving energetically efficient and dynamically stable underwater locomotion. These results suggest that distributed flapping-fin propulsion can achieve a favourable balance between force generation and energetic efficiency for both endurance-oriented and high-thrust operating modes.

The principal contributions of this work include: (i) the integration of bilateral flapping fins within a hexapod body architecture to enable distributed propulsion and stabilization, (ii) a unified validation methodology linking theory, CFD, and experimental observations, and (iii) identification of an operating regime where hydrodynamic performance and energy economy align with bio-inspired propulsion principles. The present study is intended as a hydrodynamic proof-of-concept; therefore, system-level control, full fluid–structure interaction modelling, and extended force-resolved experiments remain outside its scope. Future work will focus on adaptive fin–gait coordination, coupled fluid–structure simulations, and enhanced experimental validation using force sensors and flow diagnostics, advancing the platform toward practical applications in underwater inspection, ecological monitoring, and autonomous exploration.

## Data Availability

No datasets were generated or analysed during the current study.

## Appendix 1

Nomenclature

**Table:**

Symbol	Description	Units
A	Planform area of one fin	m^2^
C_L_	Lift coefficient	–
C_D_	Drag coefficient	–
C_p_	Pressure coefficient ((P–P_∞_)/(½ρV^2^))	–
D	Hydrodynamic drag force	N
E_d_	Energy per unit distance	J/m
f	Flapping frequency	Hz
g	Gravitational acceleration	9.81 m/s^2^
L	Hydrodynamic lift force	N
L/D	Lift-to-drag ratio	–
m	Submerged mass of robot	kg
N	Number of active fins	–
P_in_	Mechanical input power	W
P∞	Freestream (ambient) pressure	Pa
ρ	Fluid density	1000 kg/m^3^
τ	Mean driving torque per fin	N m
St	Strouhal number (fA/U)	–
T	Time-averaged thrust force	N
U	Forward swimming speed	m/s
ω	Flapping angular velocity	rad/s
µ	Dynamic viscosity of water	0.001 Pa s
Re	Reynolds number (ρVU/µ)	–

## Appendix 2

// Pseudocode: Control Logic for Spider-Inspired Underwater Robot.

BEGIN.

INITIALIZE:

Setup Arduino Uno.

Setup PCA9685 PWM drivers (2×, 16 channels each).

Configure servo pins for 6 × 3 DOF legs + 2 fins.

Configure water sensor input pin.

Initialize power modules (5 V for logic, 6 V for servos).

LOOP:

Read waterSensorValue.

IF waterSensorValue = = DRY (no water detected) THEN.

// Land mode: enable tripod gait.

CALL tripodGait().

ELSE.

// Water detected: switch to fin-based swimming.

CALL finFlappingMode().

ENDIF.

FUNCTION tripodGait():

FOR each gait cycle:

Move LeftLeg1, RightLeg2, LeftLeg3 (lift → swing → place).

Move RightLeg1, LeftLeg2, RightLeg3 (lift → swing → place).

Synchronize cycle timing with PCA9685 PWM drivers.

END FOR.

FUNCTION finFlappingMode():

Set FinLeft and FinRight to synchronous flapping.

IF turnCommand = = LEFT:

Apply phase offset (e.g., Left fin leads by 30°).

ELSE IF turnCommand = = RIGHT:

Apply phase offset (e.g., Right fin leads by 30°).

ELSE.

Maintain in-phase flapping for forward swimming.

ENDIF.

END LOOP.

END.

## Declarations

### SDG statement

This work aligns with SDG 14 (Life Below Water) by advancing energy-efficient underwater robotic propulsion for marine monitoring and inspection with minimal environmental disturbance.
